# Antiviral Role of Phenolic Compounds against Dengue Virus: A Review

**DOI:** 10.3390/biom11010011

**Published:** 2020-12-24

**Authors:** Vanessa Loaiza-Cano, Laura Milena Monsalve-Escudero, Carlos da Silva Maia Bezerra Filho, Marlen Martinez-Gutierrez, Damião Pergentino de Sousa

**Affiliations:** 1 Grupo de Investigacion en Ciencias Animales-GRICA, Universidad Cooperativa de Colombia, 680003 Bucaramanga, Colombia; vaneloaiza@gmail.com (V.L.-C.); lauramilemo@hotmail.com (L.M.M.-E.); 2 Department of Pharmaceutical Sciences, Federal University of Paraíba, CEP 58051-970 João Pessoa, PB, Brazil; carlosmaia1996@gmail.com

**Keywords:** natural products, flavonoids, tannins, phenol, medicinal plants, mosquitoes, dengue virus, viruses

## Abstract

Phenolic compounds have been related to multiple biological activities, and the antiviral effect of these compounds has been demonstrated in several viral models of public health concern. In this review, we show the antiviral role of phenolic compounds against dengue virus (DENV), the most widespread arbovirus globally that, after its re-emergence, has caused multiple epidemic outbreaks, especially in the last two years. Twenty phenolic compounds with anti-DENV activity are discussed, including the multiple mechanisms of action, such as those directed against viral particles or viral proteins, host proteins or pathways related to the productive replication viral cycle and the spread of the infection.

## 1. Introduction

Dengue virus (DENV) is an arbovirus that belongs to the family Flaviviridae, which includes Zika virus, yellow fever, Japanese encephalitis and West Nile viruses. Regarding genetic and structural characteristics, DENV is enveloped and has a spherical shape, with a positively sensed and single-stranded RNA that encodes structural proteins (capsid, membrane and envelope precursor) as well as nonstructural proteins (NS1, NS2a, NS2b, NS3, NS4a, NS4b, and NS5). In addition, DENV has four genetically distinct serotypes (DENV-1, DENV-2, DENV-3 and DENV-4) [1].

DENV infection is highly prevalent in tropical and subtropical areas. It is estimated that more than 50 million infections occur worldwide each year, and there are more than 2.5 billion people at risk of acquiring the infection [2]. All DENV serotypes can cause symptomatic infections, ranging from mild flu-like syndrome to more severe symptoms, such as coagulopathies and increased vascular permeability that can culminate in dengue hemorrhagic fever and hypovolemic shock [3]. DENV infection progresses to a severe form in only 1% of cases; however, the mortality in these cases is greater than 20% [4]. Thus, the search for molecules that have biological activity against DENV has become relevant.

Phenolic compounds are secondary metabolites produced mainly by plants. These compounds are chemically characterized by having one or more aromatic rings attached to at least one hydroxyl substituent, and it is estimated that more than 8000 different phenolic compounds have already been identified [5]. Phenolic compounds are ubiquitous in nature and have already been isolated from several plant families, including Sapindaceae [6], Vitaceae [7], Zygophyllaceae [8], Rubiaceae [9], Crassulaceae [10], Punicaceae [11], and Fabaceae [12]. They have strong antioxidant activity due to the presence of phenolic hydroxyls that give them the ability to neutralize several free radicals through the donation of hydrogen atoms, generating more stable and less toxic molecules [13,14,15]. In addition, studies indicate that these compounds have anticancer [16], anti-inflammatory [17], antibacterial [18], antifungal [19] and antiviral [20] properties.

Among the phenolic compounds that have antiviral activity are epigallocatechin gallate, which inhibits hepatitis B virus, influenza A and chikungunya virus [21,22,23]; curcumin, which is bioactive against hepatitis C virus and human immunodeficiency virus [24,25]; resveratrol, which protects against Middle East respiratory syndrome coronavirus and severe acute respiratory syndrome coronavirus 2 [26,27]; nordihydroguaiaretic acid, which protects against Zika virus and West Nile virus [28]; and punicalagin, which inhibits herpes simplex virus [29].

**Figure 1:**
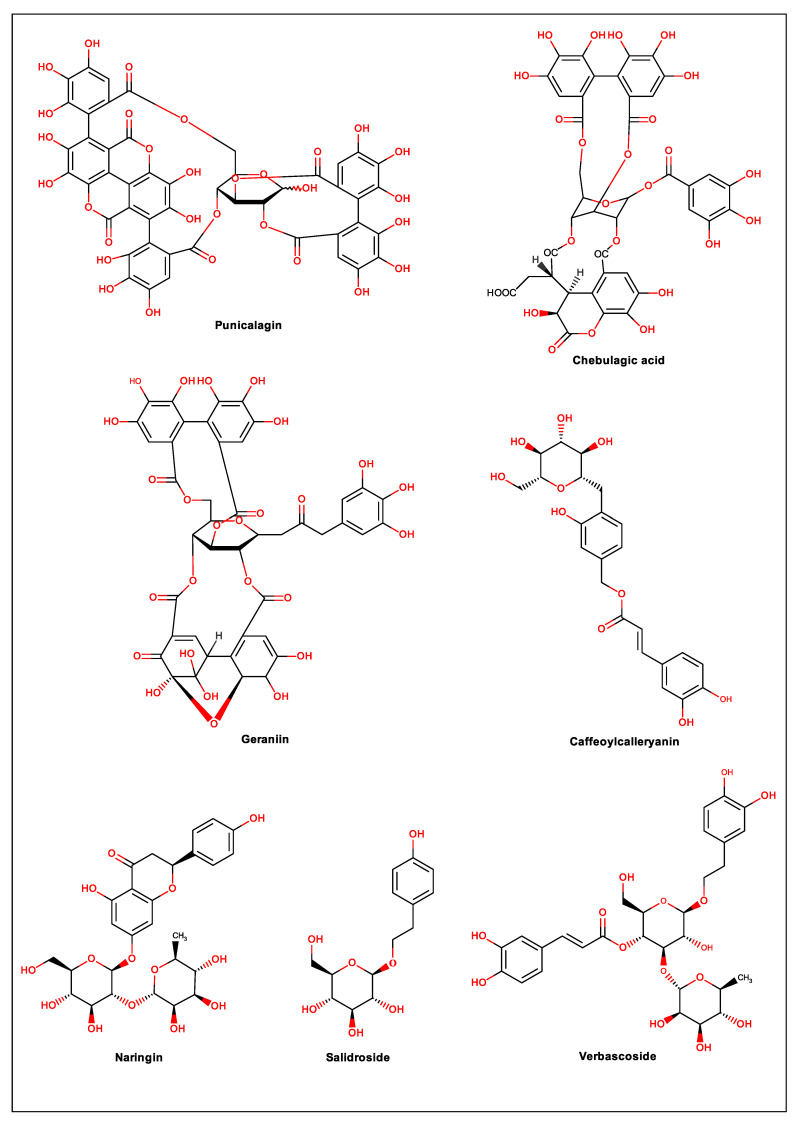
Chemical structure of heteroside phenolic compounds.

**Figure 2:**
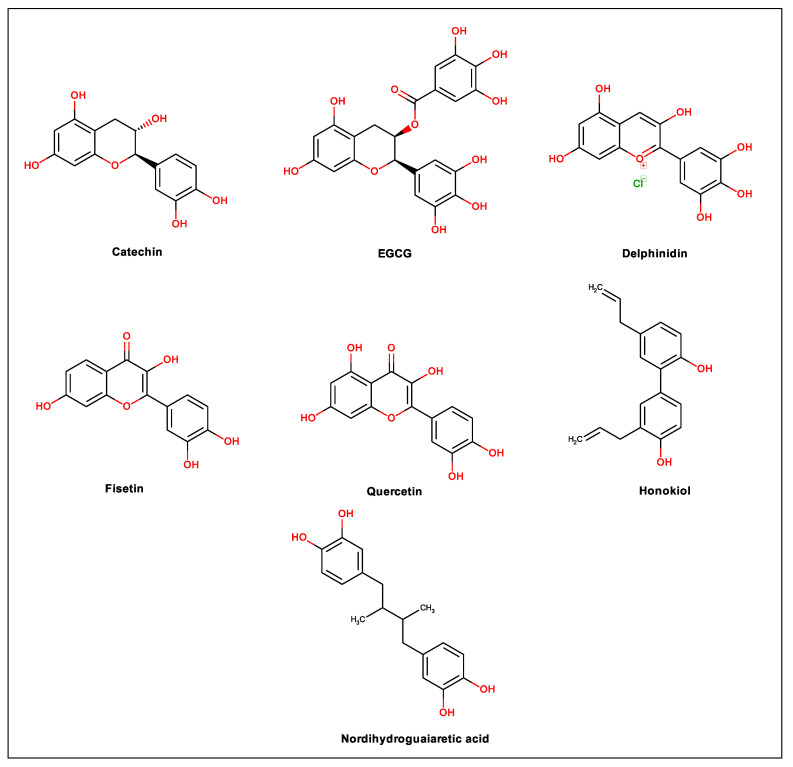
Chemical structure of flavonoids, phenylpropanoids and derivatives.

**Figure 3:**
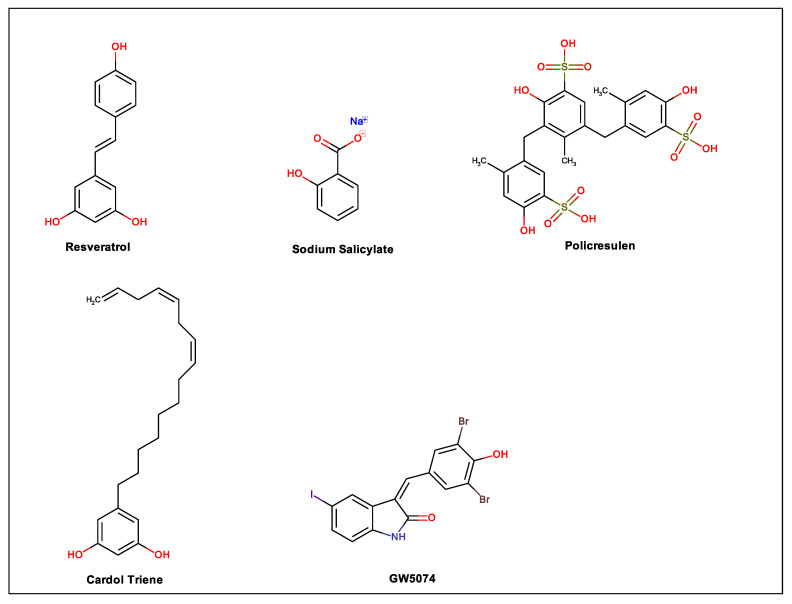
Other types of phenolic compounds.

For years, a wide variety of natural products have been the source for drug discovery due to their various structural characteristics. In this sense, compounds that have at least one phenolic group in their molecular structure represent great diversity, and most of them, including flavonoids, tannins, lignans and phenolic acids, are responsible for the antioxidant properties of many plants [20]. Oxidative stress induced by viruses is well established. This disorder interferes with the body′s important metabolic processes in addition to participating in the replication of the virus [30]. Therefore, antioxidant phenolic compounds can be interesting pharmacological tools against several types of viruses. Thus, in the present review, 20 phenolic compounds were selected, and their actions against dengue virus and mechanisms of action are discussed. Figure 1 illustrates the heteroside phenolic compounds discussed in this study; Figure 2 shows the flavonoids, phenylpropanoids and derivatives, while Figure 3 contains other types of phenolic compounds.

## 2. Geraniin

Geraniin, an ellagitannin compound, has been obtained from different plants in multiple places principally in Asia, such as *Geranuim thunbergii* Siebold ex Lindl. & Paxton (Geraniaceae) from Japan [31] and *Nephelium lappaceum* L. (Sapindaceae) [32] from Malaysia; it is the main polyphenolic component in both species and is also found in *Dimocarpus longan* Lour. and *Euphoria longan* (Lour.) Steud. from Thailand [33] and in *Phyllanthus myrtifolius* (Wight) Mull. Arg. and *P. urinaria* L. (Euphorbiaceae) from Taiwan [34], among others [33,35].

This compound has been related to multiple biological effects, such as immunomodulation induced by NF-*κ*B activation and downregulation of Mcl-1 expression to suppress ovarian cancer growth [36], anti-apoptosis effects caused by radiation damage and enhanced antioxidant enzymes in Chinese hamster lung fibroblasts (V79-4), a Hps90 ATPase inhibitor [37] and anti-*Plasmodium falciparum* in vitro (IC_50_: 11.74 µM) [36]. Antiviral in vitro effects of geraniin have also been reported against enterovirus 71 (EV71) (IC_50_: 10 μg/mL) [38], herpes simplex virus type 2 (HSV-2) (IC_50_: 18.4 ± 2.0 µM) [39], human immunodeficiency virus (HIV) (IC_50_: 0.48–6.28 μg/mL by multiple mechanisms of action) [40], hepatitis B (HBV) (200 μg/mL, inhibition of HBsAg and HBeAg secretion, 85.8 ± 7.3% and 63.7 ± 6.8, respectively) [41], Epstein–Barr virus (EBV) (IC_50_: 15.7 µM, inhibition of DNA polymerase) [42], and hepatitis C virus (HCV) (IC_50_: 8.91 µM, inhibition of NS3-4A protease) [43].

### Anti-DENV Effect of Geraniin

Due to its antiviral properties, its effect on DENV-2 infection has been studied. The antiviral effect was evaluated using a cocktail compound of four aqueous extracts of different species of local Malaysian medicinal plants (*Phyllanthus* spp.: *P. amarus* Schum. & Thonn., *P. niruri* L., *P. urinaria* and *P. watsonii* Airy Shaw) through three different strategies (pre-, trans- and post-treatment) onto confluent VERO cell monolayers. For all treatment strategies, the cultures were incubated for 24, 48 and 72 h. An antiviral effect was found in the trans-treatment (possible effect on viral particles) and pretreatment (possible effect on cellular proteins) strategies. Using a protein profiling assay, the study also demonstrated that pretreatment with *Phyllanthus* altered several cellular proteins involved in biological processes, including viral entry, viral transcription and translation regulation, cytoskeletal assembly, and cellular metabolism. Several bioactive compounds were identified in the pool, including gallic acid, syringin, corilagin, and geraniin, but the latter constituted the greatest amount in the extract. However, these components together could have had a synergistic anti-DENV effect in this study [44].

On the other hand, a study in VERO cells showed that polyphenol geraniin obtained from the bark of the *Nephelium lappaceum* L. plant had a dose-dependent virucidal effect (*trans*-treatment), with an IC_50_ of 1.75 μM. on DENV-2 [45]. Additionally, through a viral attachment assay, this polyphenol at a concentration of 26.3 μM inhibited 100% of the formation of infectious viral particles, but when its effect was evaluated after the internalization of the virus, the inhibition was only 40%. Based on these results, an in silico study was performed by molecular docking, demonstrating that the binding affinity of geraniin to domain III of the viral envelope protein is favorable, with a free energy binding of −9.8 kcal/mol. Finally, a recombinant rE-DIII protein was produced, and a competitive binding ELISA assay was performed to demonstrate that geraniin binds to this domain, avoiding viral particle adhesion to its cellular receptor [45]. All of these findings allow us to conclude that the antiviral effect of geraniin is associated with the inhibition of early steps of virus replication [45].

The anti-DENV effect of geraniin has also been demonstrated in vivo using a model of immunodeficient BALB/c mice, which develop liver damage due to infection with DENV-2 [46]. The study demonstrated that polyphenol geraniin reduced viremia when administered to mice 72 h after infection (hpi) at a concentration of 131.30 μM prepared in 100 μL PBS. Additionally, histopathology showed that treatment with geraniin 24 h prior to infection could prevent severe liver damage caused by DENV-2 [47].

## 3. Chebulagic Acid and Punicalagin

Bioactive polyphenol compounds, such as chebulagic acid and punicalagin, are also hydrolyzable tannins, such as geraniin [48], which was previously referenced in this review. Both chebulagic acid and punicalagin are simple ellagitannins that can cooccur in *Terminalia* species, but 1C4-glucopyranose core plus chebuloyl group compounds, such as chebulagic acid, have been found in the *Geranium* and *Euphorbia* genera [49], while punicalagin is more related to *Punica granatum* L., the pomegranate, where it was first isolated [49,50].

The multiple biological activities related to these two compounds are anti-inflammatory effects [51,52], growth inhibition [53], antimicrobial [54,55] and antiviral activity. The anti-HCV effect of chebulagic acid has been shown by NS3-4A protease and RNA replication inhibition (IC_50_: 9.03 µM and 22.25 ± 8.70), with higher selectivity index (SI) than geraniin in Huh 7.5 cells (4.7 vs. 1.9) [43]. In contrast, chebulagic acid inhibited EBV DNA polymerase *α* but at a higher concentration than geraniin (IC_50_: 18.6 µM) [42]. Additionally, its anti-influenza A virus (IAV) effects, as a neuraminidase inhibitor, have been probed, even for oseltamivir-resistant IAV, showing viral release inhibition (IC_50_s of 1.36 μM) but no activity on other steps of the viral cycle, such as entry or RNA replication [56]. In contrast, chebulagic acid and punicalagin inhibited HSV type 1 (HSV-1) entry and spread by acting as GAG-competitors (EC_50:_ 17.02 ± 2.82 and 10.25 ± 1.13; SI: 18.62 and 31.11, respectively) [57], as in human cytomegalovirus (HCMV) (EC_50:_ 25.50 ± 1.51; 16.76 ± 0.88), measles virus (MV) (EC_50:_ 34.42 ± 4.35; 25.49 ± 2.94), respiratory syncytial virus (RSV) (EC_50:_ 0.38 ± 0.05; 0.54 ± 0.04) and HCV (EC_50:_ 12.16 ± 2.56; 16.72 ± 2.55) in HeLa, CHO-SLAM, HEp-2 and Huh-7.5 cells, respectively [58]. Therefore, their antiviral mechanism of action is dependent on the viral model used.

### Anti-DENV Effect of Chebulagic Acid and Punicalagin

Both molecules were evaluated against DENV-2 strain 16,681 infection in HeLa, VERO, A549 and HEp-2 cells. The effectiveness of these compounds at different concentrations (1–10 and 100 µg/mL) was confirmed when prechilled monolayers at 4 °C were cotreated with DENV-2 at a multiplicity of infection (MOI) of five, at the same time demonstrating their effectiveness against infection. In this sense, there was a significant inhibition of viral particle adhesion and fusion to the cell membrane [58]. The virus seems to bind to tannins, avoiding cellular receptors, likely with a similar mechanism of action as other viral models previously referenced.

## 4. Flavonoids

The flavanols catechin and epigallocatechin gallate (EGCG) and delphinidin and the flavanone naringin and the flavanols quercetin and fisetin are derived from the phenylpropanoid metabolic pathway [59,60] and belong to the large family of flavonoid compounds derived from shikimic acid metabolism.

Flavonoid compounds have been one of the most studied secondary plant metabolites in different health disorders. The phenolic hydroxyl groups present on the B ring are related to their antioxidant activity [61], which could be associated with the wide spectrum of pharmacological activities of this group, including anti-inflammatory and antiallergic [62,63], neuroprotective [61,64], hepatoprotective [65], nephroprotective [66], anticancer [60,67] and antimicrobial [68,69,70] effects. Additionally, many flavonoid compounds have been related to antiviral activity against many viruses, such as HIV, HSV, influenza virus (IV), RSV, severe acute respiratory syndrome coronavirus (SARS-CoV), measles, and rotavirus [71]. This demonstrates that flavonoids could be one of the most active compounds against different types of viruses, with multiple mechanisms of action, such as the inhibition of adsorption, virus entry, virus binding, RTase, integrase, protease, replication inhibiting DNA and RNA polymerases, and protein complex formation [71].

Specifically, quercetin showed activity against HBV by inhibiting mRNA in HuS-E/2 cells (50 µM: approx. 40%) [72] and HBsAg secretion (36.1 ± 7.6%), but not HBeAg, in HepG2 2.2.15 cells (25 μg/mL) [41], against murine *betacoronavirus* and mouse hepatitis virus (MHV) CCL9.1 cells at low SI (IC_50_ 125.0 μg/mL; SI: 0.93) [73], against enterovirus 71 (EV71) strain Wuhan/3018/2010 in RD cells (50 μM) [74], and against canine distemper virus (CDV) in VERO cells. However, naringenin [75], the biosynthetic precursor of naringin, did not [76].

Quercetin inhibited the entry of three strains of IAV into MDCK cells (IC_50_: 7.756 ± 1.097, 6.225 ± 0.467, and 2.738 ± 1.931 μg/mL for strains A/Puerto Rico/8/34 (H1N1), A/FM-1/47/1 (H1N1), and A/Aichi/2/68 (H3N2), respectively). In the same cell model, posttreatment with quercetin and catechin hydrate, compounds present in bioactive extracts of *Aloe vera* L. (25 μg/mL), inhibited M2 viral mRNA synthesis and M2 protein expression of IAV (H1N1) strain A/PR/8/34 at an MOI of 1. An extract that contains both compounds inhibits autophagy induced by IAV infection [77]. Quercetin also stimulates Na^+^–K^+^–2Cl^−^ cotransporter 1 (NKCC1) due to its chemical structure [78], and these compounds could have activity as direct antivirals as well as a broad spectrum against other viruses.

On the other hand, other flavonoid compounds, such as fisetin, have Enterovirus A71 (EV-A71)-3C protease inhibition activity in HeLaG3CwtR cells (CI_50_: 142.8 ± 0.7 µM) and can inhibit replication (84.5 ± 0.3 µM) [79]. Naringin inhibits herpes simplex type 1 (HSV-1) (cytopathic effect (CPE) inhibitory concentration: 1.6 µg/mL), parainfluenza type-3 (PI-3) (cytopathic effect (CPE) inhibitory concentration: 0.2 µg/mL) [80], and rotavirus in MA-104 cells (IC_50_: 25 µM); catechin inhibits HBV-mRNA in HuS-E/2 cells (50 µM: more than 40%); and EGCG inhibits HBV entry (50 μM in HuS-E/2 cells) [72], cccDNA, replicative intermediates of DNA (100 μM: 72.4% and 71.8%, respectively) and HBV-HBeAg (IC_50_ of 39.4 μM) in HepG2.117 cells, but not HBsAg [81]. This is in contrast to other studies showing inhibitions above 90% in HepG2-N10 cells of both Ag at 100 μM [22]. It also has a modulatory effect on cellular processes that affect the HBV viral cycle, such as autophagy, which is necessary for replication [82] and transcription in HepG2 cells [22]. Additionally, EGCG inhibits vesicular stomatitis virus (VSV), IAV, HCV, Sindbis virus (SIN), reovirus (RV), HSV-1, HSV-2, murine cytomegalovirus (mCMV), vaccinia virus (VACV) and adenovirus type 5 (AdV) in VERO or MDCK cells (EC_50_, 3.3, 7.3 to 40.1, 2.6, 15.8, 4.3, 0.1, 2.6, 5.4, 7.1 to 7.7, and 17.7 μM, respectively) [83]. Inhibition of HIV, human T-cell lymphotropic virus (HTLV), HCV, Chikungunya virus (CHIKV), Ebola virus (EBOV), viral hemorrhagic septicemia (VHSV), infectious hematopoietic necrosis virus (IHNV), spring viremia of carp (SVCV) and grass carp reovirus (GCRV), has also been reported [84].

### Anti-DENV Effect of Flavonoids

Due to the antiviral reports of this compound, including activity against arboviruses and flaviviruses, flavonoids also have several reported anti-DENV effects with multiple mechanisms involved. Nonneutralizing heterotypic antibodies have been documented to induce antibody-dependent potentiation (ADE) in secondary DENV infection, leading to increased entry of infectious viral particles into phagocytes, cells that produce a series of proinflammatory cytokines involved in the immune response in severe dengue pathogenesis [2]. In this context, a recent study evaluated the antiviral and immunomodulatory properties of polyphenols in U937-DC-SIGN cells (boosted or not with antibodies) infected with DENV-2 and DENV-3 at an MOI of 1. Only quercetin at 100 µM and fisetin at 40 µM showed activity (flavonoids that only differ in one hydroxyl group at carbon 5 of the A ring). Furthermore, DENV-2 induced more IL-6, IFN-γ, and IL-10 than DENV-3, but both viruses induced similar amounts of TNF-α that were downregulated by the compounds [85]. Additionally, quercetin and fisetin can also induce type 1 IFN, a cytokine mediated by the JAK-STAT route, modifying the signaling pathways involved in the innate response [86].

Additionally, the antiviral effect of quercetin has been probed in different DENV-2 strains, such as the NG strain at an MOI of 1 in VERO cells (19.2 μg/mL; SI: 34.3) with pre- and post-treatment inhibition [87]; the New Guinea C strain in BHK-21 cells but with a low selectivity index (IC_50_ 176.76 μg/mL; SI: 0.88) [73]; and the TR1751 strain with an MOI of 5 in BHK-21 cells, with inhibition percentages of 60.6% and 75.7% at concentrations of 1 µM and at 10 µM, respectively [88]. The possible antiviral effect of quercetin and fisetin has even been reviewed with in silico methods, such as molecular docking, using different DENV viral proteins as possible pharmacological targets, and both could interact with glycoprotein E, glycoprotein NS1, protease NS3 and RdRP NS5. Therefore, it was assumed that polyphenols can have several mechanisms of action that inhibit different stages of the viral replicative cycle [89,90,91]. Among six phenolic compounds, quercetin had the best favorable ligand–enzyme consensus score (CScore) of 5.95 with DENV-2 NS2B-NS3 protease [92], but it did not have the best binding energy among the other five phenolic compounds with DENV NS5 and envelope proteins [87]. The inhibition and interaction of quercetin and DENV protease as important targets [93,94] could be related to their mechanism of action.

The flavonoids naringin and catechin also inhibited DENV-2 NG at MOI 1 in VERO cells (47.9 μg/mL; SI: 13.5; and 33.7 μg/mL; SI: 24.8, respectively), especially posttreatment (64.5% and 91.8% inhibition, respectively), and only catechin at pretreatment [87]. Additionally, fisetin showed an anti-DENV-2 (NGC strain) effect in VERO cells treated previously (IC_50_: 43.12 µg/mL; SI: 5.72) and after infection (IC_50_: 55 µg/mL; SI: 4.49), with no direct virucide activity. However, naringenin, a naringin precursor, exhibited direct virucidal activity against DENV-2 (IC_50_ = 52.64 µg/mL SI < 1) [95], and the anti-adsorption effects of naringin against the DENV-2 New Guinea C strain have been probed in VERO cells (IC_50_ = 168.2 μg/mL; SI: 1.3), reducing the viral genome (25.8%; 50 μg/mL) [96].

Catechin, delphinidin, quercetin and EGCG were proven effective against DENV-2 (strain 00st-22A) at an MOI of 0.03 in VERO cells. The inhibition percentages of catechin and delphinidin were above 60%, but quercetin and EGCG showed approximately 90% inhibition at the same concentration (100 μM). In the same study, the antiviral effect of EGCG was probed against the four DENV serotypes, but not other flaviviruses (100 μM), and the possible mechanism of action showed better activity at pretreatment than at posttreatment (DENV-2 MOI of 3.60 μM) [97].

The antiviral effects of the polyphenols delphinidin and EGCG in three different flaviviruses (DENV, West Nile virus (WNV) and Zika virus (ZIKV)) were evaluated. In those studies, the infection was reduced, likely by affecting virus internalization [72] or modulating endosomal pH [82], which can affect pH-dependent viral fusion. According to these results, another study determined that both phenolic compounds, delphinidin and EGCG at 10 μM, have anti-flavivirus effects in DENV, WNV and ZIKV models when added at the first steps of infection in VERO cells. The mechanism of action could act directly on the viral particle [98]. Other flavonoid compounds have shown similar mechanisms of action, such as baicalein, which exhibits virucidal and anti-adsorption activities against DENV and Japanese encephalitis virus (JEV) [99,100].

## 5. Resveratrol

Resveratrol, a natural oligomeric stilbene, is a phytoalexin principally derived from grapes, berries, peanuts, and other plant sources as one of the defense mechanisms against infection and stress and is a widely known anti-inflammatory and antioxidant agent [101]. Oligomeric stilbenes are distributed in more than 15 families of plant species [102]. Resveratrol can be found in two stereoisomeric forms, *trans-* or *cis-3*,5,4′-trihydroxystilbene, but the *trans*-isomer changes into the *cis*-isomer in the presence of ultraviolet light. These differences could impact the different biological properties [103,104,105].

Similar to other phenolic compounds, the antiviral effect of resveratrol has been tested in models, such as IAV in MDCK cells, EBV in Raji and human B cells, HSV in VERO and MRC-5 cells, RSV in lung epithelial cells, and HIV-1 in primary peripheral blood lymphocytes [106]. The anti-flavivirus activity of resveratrol was proven against ZIKV in Huh7 cells, and the mechanisms were related to postentry and virucidal activity and adsorption inhibition (MOI 1; 80 µM) [107]. Anti-HCV activity could not be proven, and treatment with resveratrol even enhanced replication in OR6 cells [108].

### Anti-DENV Effect of Resveratrol

A recent study evaluated five natural compounds and found that, among the tested compounds, only resveratrol had an antiviral effect against DENV-2/16681 in HEK293T/17 cells, but not in HepG2 cells, after viral entry. Additionally, a dose-dependent effect was observed (EC_50_: 11.37 μM) when cells were infected at a low MOI of 0.01, while the dose-dependent effect was not evident at a higher MOI of 2 (EC_50_: 24.37 μM) [109]. However, in that study, it was not possible to elucidate a specific mechanism of action that could explain these results.

A study found that resveratrol can inhibit DENV-2 infection in Huh7 cells, inducing HMGB1 protein accumulation. Resveratrol increases the amount of nuclear HMGB1 and improves the production of genes stimulated by interferon (ISG), leading to a more efficient innate immune response inside the cell, which is crucial to restrict virus replication and infection [110]. The inhibitory effect of resveratrol on the translocation of HMGB1 outside the nucleus also suggests the possibility that treatment may negatively regulate the proinflammatory genes associated with DENV disease pathogenesis [111].

Although the mechanism of action has been more elucidated for some compounds, the question remains whether structural analogs have similar properties or whether apparently small differences in the chemical structure could modify the inhibition percentages obtained and could even have a completely different mechanism of action. For these reasons, two resveratrol analogs, PNR-4-44 and PNR-5-02, were evaluated in Huh7 cells infected with DENV-2/NG, demonstrating a reduction in the cytopathic effect of the virus in a dose-dependent manner. Furthermore, both analogs had an effect at 12 h postinfection in an addition time assay, inhibiting the viral genome but not affecting the viral polymerase [112]. The study suggests that the decrease in the viral genome can be attributed to other nonviral factors, since it is known that viral replication requires the presence of cellular molecules that can be altered in the presence of analogs [113]. Because of these factors, the cellular components that intervene in the replicative cycle of the virus offer a possible therapeutic route in which they can be considered treatment targets. Although both resveratrol phenolic analogs were shown to be active in events after virus entry, only PNR-5-02 had partial inhibition in stages prior to viral entry by inhibiting replication by 34%, indicating that it may have an additional effect on cellular factors in host cells, as reported in other studies [114].

## 6. Nordihydroguaiaretic Acid

Phenolic lignan, nordihydroguaiaretic acid (NDGA), has been isolated from leaves of *Larrea tridentate* (DC.) Coville found in Mexico and USA deserts. NGDA is approximately 5 to 10% of the leaf dry weight (80% of all phenolic compounds in the resin). The catechol rings present in their structure confer antioxidant and anti-inflammatory properties to this hypolipidemic agent [115]. NDGA is also known for its cytoprotective effects in nontumor cells and its proapoptotic activity in malignant cells. These facts make NDGA a promising antitumor compound that regulates several signaling pathways and controls cellular damage by reactive oxygen species (ROS) [116,117].

It is well known that viruses can regulate cellular metabolism and pathways to develop and improve their viral replication cycle [118]. The modulatory properties of NDGA could inhibit changes in the cell after viral infection. According to this, NDGA can inhibit HIV-Tat-regulated secreted alkaline phosphatase (IC_50_ = 20 µM) [119], DNA fragmentation by apoptosis, ROS production induced by IAV (Puerto Rico/8/34; H1N1) infection (76% in human fetal membrane chorion cells) [120], and lipid metabolic pathways necessary for HCV replication in Huh7.5.1 cells (EC_50_: 30 μM) [121]. Similar regulatory mechanisms could be related to NDGA activity against arboviruses.

### Anti-DENV Effect of NDGA

A study evaluated the effect of NDGA against DENV-2/NG and DENV-4 infection in Huh-7, U937 and VERO cells. This study concluded that posttreatment with NDGA significantly inhibited DENV replication, causing a reduction in the amount of lipid droplets (neutral lipid storage organelles involved in DENV morphogenesis that increase during infection and are necessary for exocytosis of cellular metabolites and viral proteins, such as NS1) [122]. Another study showed that treatment with NDGA (100 µM) reduced secreted DENV-NS1 in Huh-7 cells by 92%; furthermore, treatment with NDGA caused dissociation of the structural protein capsid (C) from the lipid droplets, preventing the correct assembly of the DENV viral particle [123]. The requirement of protein C binding to the periphery of lipid droplets for the assembly of the virus has been described [124]; additionally, the possible inhibition of virus assembly has already been reported with other hypolipemiant drugs, such as statins [125]. These observations may confirm that viral assembly can be affected by NDGA treatment.

Considering that flaviviruses need cellular lipids to complete the replicative cycle [126,127] and that DENV infection modulates the synthesis of cholesterol and fatty acids, when generating a lipid-rich cellular environment that is necessary for viral replication [126], compounds able to modify the metabolic pathways of lipids may be an appropriate strategy to interrupt the replicative cycle of flaviviruses.

The sterol regulatory element binding protein (SREBP) pathway is another proposed mechanism even when the antiviral effect of NDGA (10 µM or 35 µM) was demonstrated in other flaviviruses, with ZIKV and WNV at an MOI of 1 in VERO-CCL81 and HeLa3-WNV cells (cells that express the structural proteins C, prM and E of WNV) [28,128]. This shows that the same compound can inhibit several viruses and has multiple mechanisms of action, making it a broad-spectrum candidate.

## 7. Curcumin

Curcumin or diferuloylmethane, derived from the phenylpropanoid pathway, is a linear diphenylheptanoid and a tautomeric compound with enol, keto and enol–keto forms; it depends on dilution in solvents that can influence its activities [129,130].

This natural compound is present in *Curcuma* species, especially *Curcuma longa* L., and has multiple reported anti-inflammatory, antioxidant, anticarcinogenic, antiangiogenic, antiplatelet aggregation, skin regeneration, antimicrobial and antiviral properties [131]. Many of these activities have been related to cellular pathways and enzyme modulation, including the transcription factor NF-*κ*B, phospholipases, cyclo-oxygenases and lipoxygenases, metalloproteinases, superoxide dismutase, catalase, glutathione peroxidase, cytochrome P450, JNK, and MAPKs, among others [132].

The antiviral activity of curcumin has been proven against many enveloped viruses, since this compound is able to modify the lipid bilayer and influences the function of the membrane protein [133]. The curcumin antiviral effect was confirmed for HBV inhibition of mRNA in HuS-E/2 (50 µM: more than 40%) [72]; for coxsackie virus (CVB3) in HeLa cells (MOI of 10; 30 µM) [134] and JEV in Neuro2a cells (MOI of 5; 5 and 10 μM) [135], acting as a host-target antiviral agent for both of these viruses by modulating ubiquitin–proteasome system; HSV-1 in pretreated VERO cells (MOI of 1; 10 μM) [136]; HIV by different mechanisms [137]; HCV entry in Huh-7.5 cells and primary human hepatocytes (IC_50_ 8.46 ± 1.27 mM in Huh-7.5 and 12.5 µM in PHH) [138]; and arboviruses like ZIKV and CHIKV in pretreated HeLa cells, inhibiting both infectious particle and viral-RNA (MOI of 0.1; IC_50_: 1.90 and 3.89 μM, respectively) [139].

### Anti-DENV Effect of Curcumin

As described above for CVB3 and JEV, the importance of curcumin in modulating cellular systems, such as the ubiquitin–proteasome, leads to an antiviral effect. In the case of DENV, it has been described that the ubiquitin–proteasome system decreases the concentration of structural E-protein that could affect DENV infection [140]. According to this, a study concluded that curcumin at different concentrations (10, 15, and 20 μM) caused intracellular accumulation of viral proteins and promoted the accumulation of ubiquitin-conjugated proteins, causing decreased DENV infection. However, the mechanism by which this system affects the replicative cycle has not yet been established [141].

The antiviral effect of curcumin against many enveloped viruses was described above. Continuing with this, a study determined that this compound completely cleared DENV-2 and another flavivirus, JEV, during the *trans*-treatment strategy. However, the antiviral effect was not evidenced when curcumin was added to the cells after infection. Consequently, these studies concluded that curcumin can act as a direct antiviral or host-target antiviral agent [142]. Due to the broad spectrum of this phenolic compound, the structural core of curcumin could be used to develop new molecules with enhanced antiviral effects.

Despite the promising effects of curcumin, its obtainment from *Curcuma longa* is limited; moreover, the extraction of the compound in large masses is not entirely feasible, and the processes are often carried out in the presence of toxic solvents, such as methanol. The aqueous extraction of *Curcuma* was evaluated as an easier process to perform, and curcumin was found to be the major component in more than 80% of the samples, followed by two remaining analogs, demethoxycurcumin and bisdemethoxycurcumin [143]. Then, a study evaluated the inhibition of DENV-4 protease activity from the recombinant protein NS2B-NS3 and determined the water soluble extracts prepared with this acid or steviol glycosides with primary inhibitory activity against the viral protease [142]. It was concluded that the glucosides used in the aqueous extraction process, stevioside (Ste), rebaudioside A (RebA), or steviol glucosides (SG), were able to maintain the biological activities of the evaluated compounds, making the extraction process easier and less toxic to obtain compounds with promising activity. In this context, Ste, RebA, and SG showed inhibitory activity against NS2B-NS3pro of DENV4, with IC_50_ values of 14.1 ± 0.2, 24.0 ± 0.4, and 15.3 ± 0.4 μg/mL, respectively [143]. However, it is important to note that, in studies using extracts, the effect cannot be attributed to a single compound and is probably due to the result of synergy between the mixture of molecules present in the extract.

## 8. Salidroside

Salidroside, also known as rhodioloside, rhodosin, tyrosol 8-*O*-glucoside or *p*-hydroxyphenethyl glucopyranoside, is a bioactive phenolic compound tyrosine derived from *Rhodiola* genus plants [144]. One of the principal biological activities related to *Rhodiola rosea* L. and salidroside is their activity in the pathogenic conditions of the central nervous system [145], osteoarthritis rat models inhibiting synovial inflammation [146] and alleviating cartilage degeneration [147], diabetic nephropathy in rats [148] and anticancer in vitro [149].

The antiviral effect of salidroside has also been reported against RSV in HEp-2 cells (MOI of 0.01; IC_50_: 10.3 ± 1.50 μg/mL) [150] and CVB3 in vitro in myocytes and in vivo in BALB/c mice (IC_50_: 39.0 ± 1.2 mg/L; 20 and 40 mg/kg at days 7 and 14) [151].

### Anti-DENV Effect of Salidroside

Studies evaluating compounds with mechanisms of action on the immune system are important due to the immunopathological nature of DENV [152]. Among these compounds is salidroside, which has neuroprotective, anti-inflammatory and antiviral properties [153,154]. This compound is derived from the plant *Rhodiola rosea*. Anti-DENV-2 activity in vitro has been demonstrated in THP-1 cells infected with DENV-2 (MOI 3) and incubated for 48 h after infection with salidroside (166 µM). The effect was determined by evaluating DENV envelope protein expression by Western blotting, and the density ratio of viral protein and salidroside-treated cells to beta actin decreased more than ten-fold in comparison to virus-infected cells without salidroside treatment [155]. It was also postulated that the mechanism of action of salidroside is related to the increased expression of RIG-I, which specifically recognizes viral RNA [156], initiating a downstream signaling cascade that induces positive regulation of IRF-3 and IRF-7, which limit initial stages of DENV infection [157]. On the other hand, salidroside increases the expression of PKR and P-eIF2*α*, which restricts the synthesis of viral proteins, decreasing the expression of NF-*κ*B [158]. Another effect is the increase in IFN-α and NK cells observed in human peripheral blood mononuclear cells (hPBMCs), which helps reduce viral replication during the early stages of DENV infection and therefore limits subsequent pathogenesis [159]. These results indicate that the phenolic glycoside salidroside could be considered for the development of an effective therapeutic multitherapeutic agent against DENV infection [155].

## 9. Verbascoside and Caffeoylcalleryanin

Verbascosides, also known as actosides and caffeoylcalleryanins, are polyphenolic catechols. They have been isolated from multiple plant families, such as Bignoniaceae [43], Lamiaceae [160], Scrophulariaceae [161], and species such as *Arrabidaea* spp. and *Cuspidaria pulchra* (Cham.) L.G. Lohmann. These tropical plants have been used for medical purposes, such as the treatment of skin effects, leukemia, anemia, colic, and diarrhea, because of their anti-inflammatory and astringent effects [162].

The leaves of *Arrabidaea chica* (Humb. & Bonpl.) B. Verlt have antifungal and trypanocidal activities [162]. Moreover, the ethanolic extracts of *Arrabidaea samydoides* (Cham.) Sandw. leaves and stems have shown antiviral effects against HHV-1 (EC_50_ 40.6 ± 1.6 μg/mL and 218.1 ± 3.4 μg/mL, respectively), encephalomyocarditis virus (EMCV) (EC_50_ 323.4 ± 5.6 and 377.2 ± 17.7 μg/mL, respectively) and VACV (EC_50_ 37.13 ± 1.3 and 45.5 ± 2.8 μg/mL, respectively) [163].

Moreover, caffeoylcalleryanin has anti-inflammatory effects, showing a significant inhibitory effect on NF-*κ*B activity at 100 μg/mL [164]. Meanwhile, purified verbascoside has demonstrated inhibition of HSV-1 and HSV-2 in VERO cells, with a virus-dependent antiviral effect (200 μg/mL), since the viricidal effect was the principal mechanism of action for HSV-1, and entry inhibition of HSV-2 [164].

### Anti-DENV Effect of Verbascoside and Caffeoylcalleryanin

The antiviral activity of both compounds against DENV-2 was proven in VERO and LLCMK2 cells treated with caffeoylcalleryanin and verbascoside for 48 h (EC_50_: 2.8 ± 0.4 μg/mL, SI: 20.0 and 3.4 ± 0.4 μg/mL, SI: 3.8, respectively) [165], but the mechanism of action was not elucidated. However, this kind of catechol compound, such as dicaffeoylquinic acid (DCQA) and related dicaffeoyltartaric acid, *L*-chicoric acid, has been shown to be involved in HIV-RT polymerase inhibition (IC_50_: 7–107 µM and 17 µM, respectively), HIV integrase inhibition (IC_50_: 7–107 µM and 17 µM, respectively) [166], and HCV replication inhibition by 3,5-DCQA (100 µM, 53%) [167].

## 10. Sodium Salicylate

The drug sodium salicylate (NaSal) (sodium 2-hydroxybenzoate) is classified by the WHO in the ATC system N02BA04, which is other analgesics and antipyretics, salicylic acid and derivatives, a group of compounds first discovered in willow trees. The extract obtained from this tree has been used as a natural anti-inflammatory medicine for centuries. NaSal belongs to a large group of compounds known as nonsteroidal anti-inflammatory drugs (NSAIDs), exerting its mechanism of action by decreasing prostaglandin E2 by inhibiting cyclooxygenase enzyme (COX) and inhibiting NF-κB activation [168,169]. This immunomodulatory effect is related to the antiviral effect of sodium salicylate against RSV infection in A549 cells [170], CMV in human coronary artery smooth muscle cells (SMCs) (2.0 mmol/L) [171], and the flavivirus JEV in neuronal and nonneuronal cells (N18 and BHK21 cells; 5 mM) [172].

### Anti- Dengue Activity of Sodium Salicylate

The effect of sodium salicylate in cultures infected with JEV or DENV-2 at an MOI 5 in a posttreatment assay in BHK-21 and N18 cells concluded that both compounds inhibit infectious viral particles in a dose-dependent manner and block virus-induced apoptosis [173]. This inhibition is probably not mediated by blocking COX activities or NF-*κ*B activation but may involve p38 MAPK activity, which plays an essential role in apoptosis activation [174]. Although the in vitro results are promising, it should be noted that salicylates are known for antiplatelet function, a situation that can be extremely dangerous in the development of severe forms of DENV [175].

## 11. Cardol Triene

A compound obtained from the nutshell of *Anacardium occidentale* L., cardol triene (5-[(8*Z*,11*Z*)-pentadeca-8,11,14-trienyl]benzene-1,3-diol) is a phenolic lipid with three double bonds [176]. Cardol triene has been described as a potent mushroom tyrosinase inhibitor [177]. Additionally, cardol triene has antiparasitic activity against *Schistosoma mansoni* worms (IC_50_: 192.6 ± 6.0 µM) [176] and against *Trypanosoma cruzi* amastigotes (11.75 ± 0.40 µM) and trypomastigotes (IC_50_: 23.36 ± 0.12 µM) [178].

### Anti-DENV Effect of Cardol Triene

The compound cardol triene also showed in vitro anti-*DENV* activity when added to VERO cells 48 h before the infection; it was able to inhibit cell membrane fusion with the viral envelope protein of DENV-2/NG (10 µM; MOI of 1). The results also showed that the major inhibition of intracellular RNA and infectious virions was observed after infection (87.00 ± 6.43% and 91.73 ± 4.53%, respectively), and even cardol triene exhibited broad spectrum inhibition against all dengue virus serotypes (DENV 1–4; EC_50_ = 5.35 µM, 7.13 µM, 8.98 µM and 8.21 µM, respectively). A predicted in silico mechanism of action by molecular docking was made. Then, it was postulated that this compound has a high affinity (energy scored between −41.44 and −50.47 kcal/mol) for the kl loops of the DENV E protein, and this complex was demonstrated to be stable by molecular dynamics (300 ns of simulation) [179].

## 12. Policresulen

Policresulen is also known as formaldehyde-*meta*-cresolsulfonic acid. This drug has been classified by those in the ATC systems as D08AE02 (dermatological, antiseptic and disinfectants) and G01AX03 (gynecological anti-infectives and antiseptics). This drug is approved by the EMA Committee for Veterinary Medicinal Products for topical use and has been commercialized in several countries as Albothyl or Lotagen^®^ as a hemostatic [180] and antimicrobial agent [181,182].

### Anti-DENV Effect of Policresulen

Viral proteases are an interesting target for the development of antivirals for DENV [183]. As a viral protease complex, NS2B/NS3 cleaves various sites of the viral polyprotein to allow the conformation of both structural and nonstructural proteins; therefore, the inhibition of NS2B/NS3 leads to a clear interruption of the replicative cycle [184].

A study performed with a recombinant viral protease found that the compound policresulen is a potent inhibitor of DENV-2 NS2B/NS3, acting as a competitive protease inhibitor, affecting its stability and efficiently decreasing virus replication [185]. To understand the interaction between this phenolic compound and the viral protease, tests were carried out based on biophysical technology, molecular coupling and directed mutagenesis. The results showed that policresulen interacts with the Gln106 and Arg133 residues of the protease through hydrogen bonds. This finding differs from previously described interactions with other DENV protease inhibitors that bind to catalytic triad residues (His51, Asp75, and Ser135), offering a new target site for the protease [186].

## 13. GW5074

GW5074 ((3*Z*)-3-[(3,5-dibromo-4-hydroxyphenyl)methylidene]-5-iodo-1*H*-indol-2-one) is a 3′ substituted indolone. This structure has been related to neuroprotective activities, since this chemical core has been used and improved, and this biological activity remains [187] and even is related to the capability of this compound to cross the blood–brain barrier (BBB). GW5074 has been reported as a potent in vitro inhibitor of the kinase c-Raf [188], but in neurons and in in vivo models, it has the opposite action, activating B-Raf and C-Raf, which are mainly responsible for the neuroprotective effect [189,190].

The principal biological effects reported for this compound are related to its capability to modulate signaling pathways [191,192], but the antiviral activity reported against poliovirus (PV) and enterovirus 71 (EV71) in RD cells (IC_50_ of 2.7 SI: 63; IC_50_: 2.0 SI: 85) was not related to c-Raf, B-Raf or IFN response [193].

### Anti-DENV Effect of GW5074

Among the articles that included the evaluation of antivirals targeting cell targets, a small molecule, GW5074, blocks the entry of RNA-dependent RNA polymerase (RdRp) into the cell nucleus [194]. VERO cells were treated with or without GW5074 (20 µM) 2 h prior to infection with DENV-2 strain NGC at an MOI of 4. The results showed a marked reduction in NS5 nuclear localization of 2an by immunofluorescence. The mechanism of action indicates that GW5074 interferes with binding to the IMPα/β1 heterodimer, a nuclear transport protein involved in the import of NS5 into the nucleus and thus with the depletion of the subsequent impact of the antiviral response by the cell [194].

## 14. Honokiol

Honokiol is a lignan biphenol derived from the shikimic acid pathway. This compound can be obtained from the *Magnolia* Tree and is regularly used for the relief of anxiety and as analgesic in Korean, Chinese and Japanese traditional medicine [195]. This compound has shown anti-inflammatory [196], antithrombotic [197], and antioxidant activities that could be used in dermatological [198], cardiac [199] and neurological disorders [200]. Honokiol also induced apoptosis and reduced the proliferation index in implanted human prostate cancer cell (PC-3) tumors in mice [201] and had antitumoral effects against angiosarcoma implanted in mice in vivo and as an angiogenesis inhibitor in vitro [202,203]. Its antimicrobial activities include reported antibacterial effects against methicillin-resistant *Staphylococcus aureus* (MRSA) [204] and as an antiviral inhibitor of HSV-1 DNA replication and virus production [205] and HCV entry, replication and protein translation (SI: 5.4) [206].

### Anti-DENV Effect of Honokiol

**Figure 4:**
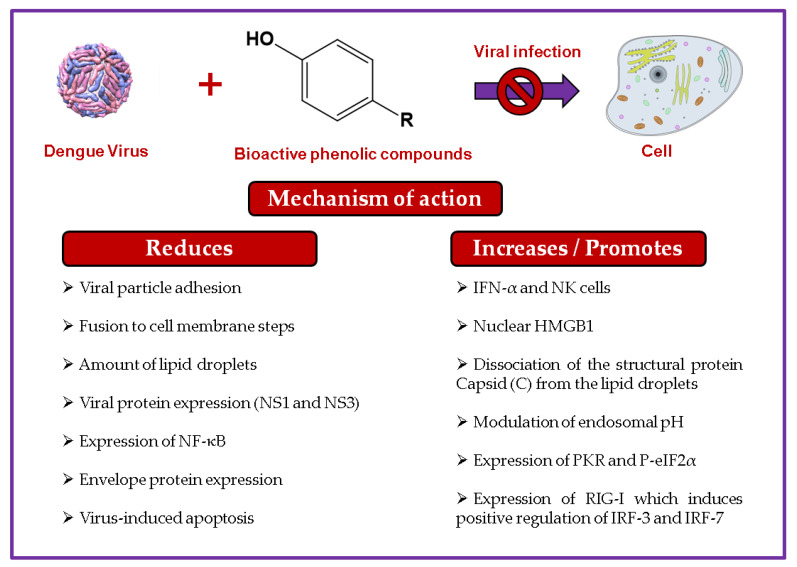
Main antiviral mechanisms of phenolic compounds against dengue virus.

DENV-2 strain PL046 infection in BHK and Huh-7 cells (MOI 0.1 and 1, respectively) was inhibited by honokiol posttreatment (48 h; 10 µM and 20 µM) by more than 90%. The possible mechanisms of action in both cellular models, BHK and Huh-7 cells treated with 10 and 20 µM honokiol, respectively, included viral protein expression reduction (NS1 and NS3; *p* < 0.001) and viral replication inhibition (intermediate, double-stranded RNA—dsARN—reduction; *p* < 0.01). Additionally, it was demonstrated that honokiol could inhibit the early steps of DENV infection, suppressing the upregulation of early endosomes, but it did not affect the attachment of the virus in Huh-7 cells (MOI of 10; 10 µM and 20 µM honokiol posttreatment) [207]. This lignan inhibits infection by different mechanisms in different viruses. Table 1 shows the anti-dengue activity of phenolic compounds, while Figure 4 illustrates the activities of these compounds against dengue virus.

## 15. Materials and Methods

The present study was carried out based on a search of the literature on phenolic compounds and dengue virus. The search, performed in the PubMed database, included studies published from 2010 until March 2020 and used the following keywords: dengue virus, phenol, polyphenol, phenol compounds, phenolic compounds, flavonoid, quercetin, tannins and lignans. Scientific publications were selected from studies published in English.

## 16. Conclusions

The results discussed in this review show the clinical potential of phenolic compounds as antiviral agents, especially against dengue virus. Some of the compounds are widely found in medicinal plants and foods or are drugs used for other clinical purposes; thus, they may have greater toxicological safety for use in humans as anti-dengue drugs. Despite the structural diversity of bioactive compounds, it is not possible to establish a structure–antiviral activity relationship. However, the presence of phenolic hydroxyl groups in chemical structures should have an important contribution to antiviral action and should be investigated for the development of synthetic derivatives with therapeutic applications against dengue infection.

## Figures and Tables

**Table 1:** Phenolic compounds with activity against dengue virus.

Compound and Structure	IUPAC Name	Experimental Model Used	IC_50_	Mechanism of Action	Reference
**Geraniin**	[(1R,7R,8S,26R,28S,29R,38R)- 1,13,14,15,18,19,20,34,35,39,39-undecahydroxy-2,5,10,23,31-pentaoxo-6,9,24,27,30,40 exaoxaoctacyclo[34.3.1.04,38.07,26.08,29.011,16.017,22.032,37]tetraconta-3,11,13,15,17,19,21,32,34,36-decaen-28-yl] 3,4,5-trihydroxybenzoate	VERO cells	8.91 µM	Possible effect on viral particle Effect on cellular proteins involved in viral replication cycle and cellular metabolisms	[44]
		VERO cells Molecular docking	1.75 μM	Dose-dependent virucidal effect Inhibition of adhesion of viral particle Possible inhibition of early steps of virus replication cycle Interference with cell receptor interaction by binding to the E-DIII protein	[45]
		BALB/c mice	1.78 μM	Viremia reduction Prevention of liver damage	[47]
**Chebulagic Acid**	2-[(4R,5S,7R,25S,26R,29S,30S,31S)-13,14,15,18,19,20,31,35,36-nonahydroxy-2,10,23,28,32-pentaoxo-5-(3,4,5-trihydroxybenzoyl)oxy-3,6,9,24,27,33-hexaoxaheptacyclo[28.7.1.04,25.07,26.011,16.017,22.034,38]octatriaconta-1(37),11,13,15,17,19,21,34(38),35-nonaen-29-yl]acetic acid	HELA, VERO, A549 and HEp-2 cells.	13.11 μM	Inhibition of viral particle adhesion and fusion to cell membrane steps Possible GAG-competitor	[58]
**Punicalagin**	(1R,35R,38R,55S)-6,7,8,11,12,23,24,27,28,29,37,43,44,45,48,49,50-heptadecahydroxy-2,14,21,33,36,39,54-heptaoxaundecacyclo[33.20.0.04,9.010,19.013,18.016,25.017,22.026,31.038,55.041,46.047,52]pentapentaconta-4,6,8,10,12,16,18,22,24,26,28,30,41,43,45,47,49,51-octadecaene-3,15,20,32,40,53-hexone	HELA, VERO, A549 and HEp-2 cells.	7.86 μM	Inhibition of viral particle adhesion and fusion to cell membrane steps Possible GAG-competitor	[58]
**Quercetin**	2-(3,4-dihydroxyphenyl)-3,5,7-trihydroxychromen-4-one	U937-DC-SIGN cells	24.5 µM	Downregulation of TNF-*α*	[85]
		Molecular docking	Unreported Unreported	In silico interaction with E, NS1, NS3 and NS5 proteins	[89,90]
		VERO cells	19.2 μg/mL	Inhibition in pre and posttreatment strategies but mechanism not completely elucidated	[87]
		BHK-21 cells	125 μg/mL	Possible virucide effect	[73]
		Molecular docking and enzymatic reaction	35.2 µM ^a^ 22.7 µM ^b^	Enzymatic inhibition of DENV-2 ^a^ and DENV-3 ^b^ NS2B-NS3 protease and in silico interaction with DENV-3 protease	[91]
		In silico	Unreported	Protease binding	[92]
		In silico; BHK-21 cells	Unreported	Protease binding; inhibition adsorption of viral particles	[88]
**Fisetin**	2-(3,4-dihydroxyphenyl)-3,7-dihydroxychromen-4-one	U937-DC-SIGN cells	7.3 µM	Downregulation of TNF-*α*	[85]
		Molecular docking	Unreported Unreported	In silico interaction with E, NS1, NS2B-NS3 and NS5 proteins	[89,90]
		VERO cells	43.12 µg/mL ^c^ 55 µg/mL ^d^ 50 µg/mL ^e^	Inhibition in pre ^c^ and posttreatment ^d^ strategies, and genome inhibition ^e^ but mechanism not completely elucidated	[95]
**Naringin**	((2S)-7-[(2S,3R,4S,5S,6R)-4,5-dihydroxy-6-(hydroxymethyl)-3-[(2S,3R,4R,5R,6S)-3,4,5-trihydroxy-6-methyloxan-2-yl]oxyoxan-2-yl]oxy-5-hydroxy-2-(4-hydroxyphenyl)-2,3-dihydrochromen-4-one)	VERO cells	47.9 μg/mL	Inhibition in posttreatment strategy but mechanism not completely elucidated	[87]
		VERO cells	168.2 μg mL	Anti-adsorption activity with reduction in RNA production	[96]
**Catechin**	(2R,3S)-2-(3,4-dihydroxyphenyl)-3,4-ddihydro-2H-chromene-3,5,7-triol	VERO cells	33.7 μg/mL	Inhibition in pre and posttreatment strategies but mechanism not completely elucidated	[87]
		VERO cells	Unreported	Mechanism not completely elucidated	[97]
**Delphinidin**	2-(3,4,5-trihydroxyphenyl)chromenylium-3,5,7-triol;chloride	VERO cells	Unreported	Mechanism not completely elucidated	[97]
**EGCG**	[(2R,3R)-5,7-dihydroxy-2-(3,4,5-trihydroxyphenyl)-3,4-dihydro-2H-chromen-3-yl] 3,4,5-trihydroxybenzoate	VERO cells	18.0 µM	Inhibition in pretreatment strategy but mechanism not completely elucidated	[97]
		VERO cells	Unreported	Directed to viral particle	[98]
**Resveratrol**	5-[(E)-2-(4-hydroxyphenyl)ethenyl]benzene-1,3-diol	HEK293T/17 cells	24.37 μM	Dose-dependent inhibition in stages after viral entry but mechanism not completely elucidated	[109]
		Huh7 cells	Unreported	Induction of HMGB1 protein accumulation Induction of interferon stimulated genes (ISG)	[110]
		Huh7 cells	8.12 nM ^f^ 7.22 nM ^g^	Inhibition of viral genome not affecting the viral polymerase (resveratrol analogs PNR-4-44 ^f^ and PNR-5-02 ^g^)	[112]
**Nordihydroguaiaretic acid**	4-[4-(3,4-dihydroxyphenyl)-2,3-dimethylbutyl]benzene-1,2-diol	Huh-7, U937 and VERO cells	Unreported	Reduction in the amount of lipid droplets; Reduction in the production of NS1; Prevention of the correct assembly of the DENV viral particle	[123]
**Curcumin**	(1E,6E)-1,7-bis(4-hydroxy-3-methoxyphenyl)hepta-1,6-diene-3,5-dione	BHK-21 or VERO cells	11.51 µM	Intracellular accumulation of viral proteins and ubiquitin-conjugated proteins but mechanism not completely elucidated	[141]
		VERO cells	Unreported	Could affect cell-membrane and viral envelope structure	[142]
**Salidroside**	(2R,3S,4S,5R,6R)-2-(hydroxymethyl)-6-[2-(4-hydroxyphenyl)ethoxy]oxane-3,4,5-triol	hPBMC, VERO and THP-1 cells	Unreported	Activation of type 1 interferons via IRF-3	[155]
**Verbascoside**	([(2R,3R,4R,5R,6R)-6-[2-(3,4-dihydroxyphenyl)ethoxy]-5-hydroxy-2-(hydroxymethyl)-4-[(2S,3R,4R,5R,6S)-3,4,5-trihydroxy-6-methyloxan-2-yl]oxyoxan-3-yl] (E)-3-(3,4-dihydroxyphenyl)prop-2-enoate)	VERO and LLCMK2 cells	3.4 μg/mL	Mechanism not completely elucidated	[165]
**Caffeoylcalleryanin**	[3-hydroxy-4-[[(2S,3R,4R,5S,6R)-3,4,5-trihydroxy-6-(hydroxymethyl)oxan-2-yl]methyl]phenyl]methyl (E)-3-(3,4-dihydroxyphenyl)prop-2-enoate	VERO and LLCMK2 cells	2.8 μg/mL	Mechanism not completely elucidated	[165]
**Sodium salicylate**	Sodium 2-hydroxybenzoate	BHK-21 and N18 cells	Unreported	Dose-dependent inhibition posttreatment but mechanism not completely elucidated	[173]
**Cardol triene**	5-[(8Z,11Z)-pentadeca-8,11,14-trienyl]benzene-1,3-diol	VERO cells	7.13 µM	Inhibition of cell membrane fusion with the viral envelope protein	[179]
**Policresulen**	2-hydroxy-3,5-bis[(4-hydroxy-2-methyl-5-sulfophenyl)methyl]-4-methylbenzenesulfonic acid	BHK-21 cells transfected with Rlu-DENV-Rep	4.99 μg/mL	Inhibition of DENV2 NS2B/NS3 protease	[185]
**GW5074**	(3Z)-3-[(3,5-dibromo-4-hydroxyphenyl)methylidene]-5-iodo-1H-indol-2-one	VERO cells	5.4 µM ^h^ 0.5 µM ^i^	Inhibition of NS5–IMPα/β1 interaction in vitro ^h^ as well as NS5 nuclear localization in infected cells; posttreatment activity ^i^	[194]
**Honokiol**	2-(4-hydroxy-3-prop-2-enylphenyl)-4-prop-2-enylphenol	BHK and Huh7 cells	10.6 µM	Inhibit early steps of DENV infection, suppressing the upregulation of early endosomes Reduce viral protein expression (NS1 and NS3) and double-stranded RNA	[207]

^a^ Enzymatic inhibition of DENV-2, ^b^ DENV-3, ^c^ Inhibition in pre, ^d^ posttreatment, ^e^ genome inhibition, ^f^ resveratrol analogs PNR-4-44, ^g^ PNR-5-02, ^h^ Inhibition of NS5–IMPα/β1 interaction in vitro, ^i^ NS5 nuclear localization in infected cells; posttreatment activity.

## Abbreviation

ADE	Antibody-dependent potentiation
AdV	Adenovirus type 5
BBB	Blood–brain barrier
CDV	Canine Distemper Virus
CHIKV	Chikungunya virus
COX	Cyclo-oxygenase enzyme
CPE	Cytopathic effect
Cscore	Ligand–enzyme consensus score
CVB3	Coxsackie virus
DENV	Dengue virus
EBOV	Ebola virus
EBV	Epstein Barr virus
EGCG	Epigallocatechin gallate
EMCV	Encephalomyocarditis virus
EVA71	Enterovirus A71
GCRV	Grass carp reovirus
HBV	Hepatitis B
HCMV	Human cytomegalovirus
HCV	Hepatitis C virus
HIV	Human immunodeficiency virus
HSV	Herpes simplex virus
HTLV	Human t-cell lymphotropic virus
IAV	Influenza A virus
IHNV	Infectious hematopoietic necrosis virus
ISG	Stimulated by interferon
IV	Influenza virus
JEV	Japanese encephalitis virus
mCMV	Murine cytomegalovirus
MHV	Mouse hepatitis virus
MOI	Multiplicity of infection
MRSA	Methicillin-resistant Staphylococcus aureus
MV	Measles virus
NaSal	Sodium salicylate
NDGA	Nordihydroguaiaretic acid
NKCC1	Na^+^–K^+^–2Cl^−^ cotransporter 1
NSAIDs	Nonsteroidal anti-inflammatory drugs
PI-3	Parainfluenza type-3
PV	Poliovirus
RebA	Rebaudioside A
ROS	Reactive oxygen species
RSV	Respiratory syncytial virus
RV	Reovirus
SARS-CoV	Severe acute respiratory syndrome-coronavirus
SG	Steviol glucosides
SIN	Sindbis virus
SREBP	Sterol regulatory element binding protein
Ste	Extraction process stevioside
SVCV	Spring viraemia of carp
VACV	Vaccinia virus
VHSV	Viral hemorrhagic septicemia
VSV	Vesicular stomatitis virus
WNV	West Nile virus
ZIKV	Zika virus

## References

[1] Uno N., Ross T.M. (2018). Dengue virus and the host innate immune response. Emerg. Microbes Infect..

[2] Martina B.E.E., Koraka P., Osterhaus A.D.M.E. (2009). Dengue virus pathogenesis: An integrated view. Clin. Microbiol. Rev..

[3] Sandoval E., Téllez Y., Harris E., Videa E., Amador J.J., Gonzalez A., Pérez L., Campo L.A., Pérez M.L., Cuadra R. (2000). Clinical, epidemiologic, and virologic features of dengue in the 1998 epidemic in Nicaragua. Am. J. Trop. Med. Hyg..

[4] Screaton G., Mongkolsapaya J., Yacoub S., Roberts C. (2015). New insights into the immunopathology and control of dengue virus infection. Nat. Rev. Immunol..

[5] Rodríguez-Pérez C., Segura-Carretero A., del Mar Contreras M. (2019). Phenolic compounds as natural and multifunctional anti-obesity agents: A review. Crit. Rev. Food Sci. Nutr..

[6] Thitilertdecha N., Teerawutgulrag A., Kilburn J.D., Rakariyatham N. (2010). Identification of major Phenolic compounds from *Nephelium lappaceum* L. and their antioxidant activities. Molecules.

[7] Sáez V., Pastene E., Vergara C., Mardones C., Hermosín-Gutiérrez I., Gómez-Alonso S., Gómez M.V., Theoduloz C., Riquelme S., von Baer D. (2018). Oligostilbenoids in Vitis vinifera L. Pinot Noir grape cane extract: Isolation, characterization, in vitro antioxidant capacity and anti-proliferative effect on cancer cells. Food Chem..

[8] Batallán G., Torre R., Flores F., Konigheim B., Ludueña-Almeida F., Tonn C., Contigiani M., Almirón W. (2013). Larvicidal activity of crude extracts from Larrea cuneifolia (Zygophyllaceae) and of its metabolite nordihydroguaiaretic acid against the vector Culex quinquefasciatus (Diptera: Culicidae). Rev. Soc. Bras. Med. Trop..

[9] Wolff T., Berrueta L.A., Valente L.M.M., Barboza R.S., Neris R.L.S., Guimarães-Andrade I.P., Assunção-Miranda I., Nascimento A.C., Gomes M., Gallo B. (2019). Comprehensive characterisation of polyphenols in leaves and stems of three anti-dengue virus type-2 active Brazilian *Faramea* species (Rubiaceae) by HPLC-DAD-ESI-MS/MS. Phytochem. Anal..

[10] Yu H., He Y., She Y., Wang M., Yan Z., Ren J.H., Cao Z., Shao Y., Wang S., Abd El-Aty A.M. (2019). Preparation of molecularly imprinted polymers coupled with high-performance liquid chromatography for the selective extraction of salidroside from Rhodiola crenulata. J. Chromatogr. B.

[11] Barbieri M., Heard C.M. (2019). Isolation of punicalagin from Punica granatum rind extract using mass-directed semi-preparative ESI-AP single quadrupole LC-MS. J. Pharm. Biomed. Anal..

[12] Çevik D., Kan Y., Kırmızıbekmez H. (2019). Mechanisms of action of cytotoxic phenolic compounds from Glycyrrhiza iconica roots. Phytomedicine.

[13] Cianciosi D., Forbes-Hernández T., Afrin S., Gasparrini M., Reboredo-Rodriguez P., Manna P., Zhang J., Bravo Lamas L., Martínez Flórez S., Agudo Toyos P. (2018). Phenolic compounds in honey and their associated health benefits: A review. Molecules.

[14] Xiang J., Apea-Bah F.B., Ndolo V.U., Katundu M.C., Beta T. (2019). Profile of phenolic compounds and antioxidant activity of finger millet varieties. Food Chem..

[15] Rice-Evans C.A., Miller N.J. (1996). Antioxidant activities of flavonoids as bioactive components of food. Biochem. Soc. Trans..

[16] Saud S.M., Li W., Morris N.L., Matter M.S., Colburn N.H., Kim Y.S., Young M.R. (2014). Resveratrol prevents tumorigenesis in mouse model of Kras activated sporadic colorectal cancer by suppressing oncogenic Kras expression. Carcinogenesis.

[17] Boakye Y.D., Agyare C., Abotsi W.K.M., Ayande P.G., Ossei P.P.S. (2016). Anti-inflammatory activity of aqueous leaf extract of Phyllanthus muellerianus (Kuntze) Exell. and its major constituent, geraniin. J. Ethnopharmacol..

[18] Izui S., Sekine S., Maeda K., Kuboniwa M., Takada A., Amano A., Nagata H. (2016). Antibacterial activity of curcumin against Periodontopathic bacteria. J. Periodontol..

[19] Andrade J.T., Fantini de Figueiredo G., Cruz L.F., Eliza de Morais S., Souza C.D.F., Pinto F.C.H., Ferreira J.M.S., de Freitas Araújo M.G. (2019). Efficacy of curcumin in the treatment of experimental vulvovaginal candidiasis. Rev. Iberoam. Micol..

[20] Zhang X.-L., Guo Y.-S., Wang C.-H., Li G.-Q., Xu J.-J., Chung H.Y., Ye W.-C., Li Y.-L., Wang G.-C. (2014). Phenolic compounds from Origanum vulgare and their antioxidant and antiviral activities. Food Chem..

[21] Weber C., Sliva K., von Rhein C., Kümmerer B.M., Schnierle B.S. (2015). The green tea catechin, epigallocatechin gallate inhibits chikungunya virus infection. Antiviral Res..

[22] Xu J., Gu W., Li C., Li X., Xing G., Li Y., Song Y., Zheng W. (2016). Epigallocatechin gallate inhibits hepatitis B virus via farnesoid X receptor alpha. J. Nat. Med..

[23] Yang Z.-F., Bai L.-P., Huang W., Li X.-Z., Zhao S.-S., Zhong N.-S., Jiang Z.-H. (2014). Comparison of in vitro antiviral activity of tea polyphenols against influenza A and B viruses and structure–activity relationship analysis. Fitoterapia.

[24] Prasad S., Tyagi A.K. (2015). Curcumin and its analogues: A potential natural compound against HIV infection and AIDS. Food Funct..

[25] Kim K., Kim K.H., Kim H.Y., Cho H.K., Sakamoto N., Cheong J. (2010). Curcumin inhibits hepatitis C virus replication via suppressing the Akt-SREBP-1 pathway. FEBS Lett..

[26] Horne J.R., Vohl M.-C. (2020). Biological plausibility for interactions between dietary fat, resveratrol, *ACE2*, and SARS-CoV illness severity. Am. J. Physiol. Metab..

[27] Lin S.-C., Ho C.-T., Chuo W.-H., Li S., Wang T.T., Lin C.-C. (2017). Effective inhibition of MERS-CoV infection by resveratrol. BMC Infect. Dis..

[28] Merino-Ramos T., Jiménez de Oya N., Saiz J.-C., Martín-Acebes M.A. (2017). Antiviral activity of Nordihydroguaiaretic acid and its derivative Tetra-O-Methyl Nordihydroguaiaretic acid against West Nile Virus and Zika Virus. Antimicrob. Agents Chemother..

[29] Houston D.M.J., Bugert J.J., Denyer S.P., Heard C.M. (2017). Potentiated virucidal activity of pomegranate rind extract (PRE) and punicalagin against Herpes simplex virus (HSV) when co-administered with zinc (II) ions, and antiviral activity of PRE against HSV and aciclovir-resistant HSV. PLoS ONE.

[30] Krylova N.V., Popov A.M., Leonova G.N. (2016). Antioxidants as potential antiviral agents for Flavivirus Infections. Antibiot. khimioterapiia = Antibiot. Chemoterapy [sic].

[31] Yamada H., Wakamori S., Hirokane T., Ikeuchi K., Matsumoto S. (2018). Structural revisions in Natural Ellagitannins. Molecules.

[32] Elendran S., Wang L.W., Prankerd R., Palanisamy U.D. (2015). The physicochemical properties of geraniin, a potential antihyperglycemic agent. Pharm. Biol..

[33] Sudjaroen Y., Hull W.E., Erben G., Würtele G., Changbumrung S., Ulrich C.M., Owen R.W. (2012). Isolation and characterization of ellagitannins as the major polyphenolic components of Longan (Dimocarpus longan Lour) seeds. Phytochemistry.

[34] Wang X., Chen Z., Li X., Jiang Z., Zhao Y., Ping F. (2017). Geraniin suppresses ovarian cancer growth through inhibition of NF-κB activation and downregulation of Mcl-1 expression. J. Biochem. Mol. Toxicol..

[35] Lipińska L., Klewicka E., Sójka M. (2014). The structure, occurrence and biological activity of ellagitannins: A general review. Acta Sci. Pol. Technol. Aliment..

[36] Ndjonka D., Bergmann B., Agyare C., Zimbres F.M., Lüersen K., Hensel A., Wrenger C., Liebau E. (2012). In vitro activity of extracts and isolated polyphenols from West African medicinal plants against Plasmodium falciparum. Parasitol. Res..

[37] Vassallo A., Vaccaro M.C., De Tommasi N., Dal Piaz F., Leone A. (2013). Identification of the plant compound Geraniin as a novel Hsp90 inhibitor. PLoS ONE.

[38] Yang Y., Zhang L., Fan X., Qin C., Liu J. (2012). Antiviral effect of geraniin on human enterovirus 71 in vitro and in vivo. Bioorg. Med. Chem. Lett..

[39] Yang C.-M., Cheng H.-Y., Lin T.-C., Chiang L.-C., Lin C.-C. (2007). The in vitro activity of geraniin and 1,3,4,6-tetra-O-galloyl-β-d-glucose isolated from Phyllanthus urinaria against herpes simplex virus type 1 and type 2 infection. J. Ethnopharmacol..

[40] Notka F., Meier G., Wagner R. (2003). Inhibition of wild-type human immunodeficiency virus and reverse transcriptase inhibitor-resistant variants by Phyllanthus amarus. Antivir. Res..

[41] Li J., Huang H., Feng M., Zhou W., Shi X., Zhou P. (2008). In vitro and in vivo anti-hepatitis B virus activities of a plant extract from *Geranium carolinianum* L.. Antivir. Res..

[42] Chen Liu K.C.S., Lin M.-T., Lee S.-S., Chiou J.-F., Ren S., Lien E.J. (1999). Antiviral Tannins from two Phyllanthus species. Planta Med..

[43] Li Y., Yu S., Liu D., Proksch P., Lin W. (2012). Inhibitory effects of polyphenols toward HCV from the mangrove plant *Excoecaria agallocha* L.. Bioorg. Med. Chem. Lett..

[44] Lee S.H., Tang Y.Q., Rathkrishnan A., Wang S.M., Ong K.C., Manikam R., Payne B.J., Jaganath I.B., Sekaran S.D. (2013). Effects of cocktail of four local Malaysian medicinal plants (Phyllanthus spp.) against dengue virus 2. BMC Complement Altern. Med..

[45] Abdul Ahmad S.A., Palanisamy U.D., Tejo B.A., Chew M.F., Tham H.W., Syed Hassan S. (2017). Geraniin extracted from the rind of Nephelium lappaceum binds to dengue virus type-2 envelope protein and inhibits early stage of virus replication. Virol. J..

[46] Paes M.V., Pinhão A.T., Barreto D.F., Costa S.M., Oliveira M.P., Nogueira A.C., Takiya C.M., Farias-Filho J.C., Schatzmayr H.G., Alves A.M.B. (2005). Liver injury and viremia in mice infected with dengue-2 virus. Virology.

[47] Abdul Ahmad S.A., Palanisamy U.D., Khoo J.J., Dhanoa A., Syed Hassan S. (2019). Efficacy of geraniin on dengue virus type-2 infected BALB/c mice. Virol. J..

[48] Chen F., Tang Q., Ma H., Bian K., Seeram N.P., Li D. (2019). Hydrolyzable Tannins are iron chelators that inhibit DNA repair enzyme ALKBH2. Chem. Res. Toxicol..

[49] Yoshida T., Amakura Y., Yoshimura M. (2010). Structural features and biological properties of Ellagitannins in some plant families of the order Myrtales. Int. J. Mol. Sci..

[50] Heber D. (2011). Pomegranate ellagitannins. Herbal Medicine: Biomolecular and Clinical Aspects.

[51] Reddy D.B., Reddanna P. (2009). Chebulagic acid (CA) attenuates LPS-induced inflammation by suppressing NF-κB and MAPK activation in RAW 264.7 macrophages. Biochem. Biophys. Res. Commun..

[52] Dell’Agli M., Galli G.V., Bulgari M., Basilico N., Romeo S., Bhattacharya D., Taramelli D., Bosisio E. (2010). Ellagitannins of the fruit rind of pomegranate (Punica granatum) antagonize in vitro the host inflammatory response mechanisms involved in the onset of malaria. Malar. J..

[53] Adaramoye O., Erguen B., Nitzsche B., Höpfner M., Jung K., Rabien A. (2017). Punicalagin, a polyphenol from pomegranate fruit, induces growth inhibition and apoptosis in human PC-3 and LNCaP cells. Chem. Biol. Interact..

[54] Silva O., Viegas S., de Mello-Sampayo C., Costa M.J.P., Serrano R., Cabrita J., Gomes E.T. (2012). Anti-Helicobacter pylori activity of Terminalia macroptera root. Fitoterapia.

[55] Endo E.H., Garcia Cortez D.A., Ueda-Nakamura T., Nakamura C.V., Dias Filho B.P. (2010). Potent antifungal activity of extracts and pure compound isolated from pomegranate peels and synergism with fluconazole against Candida albicans. Res. Microbiol..

[56] Li P., Du R., Wang Y., Hou X., Wang L., Zhao X., Zhan P., Liu X., Rong L., Cui Q. (2020). Identification of Chebulinic Acid and Chebulagic Acid as novel Influenza Viral Neuraminidase inhibitors. Front. Microbiol..

[57] Lin L.-T., Chen T.-Y., Chung C.-Y., Noyce R.S., Grindley T.B., McCormick C., Lin T.-C., Wang G.-H., Lin C.-C., Richardson C.D. (2011). Hydrolyzable Tannins (Chebulagic Acid and Punicalagin) target viral Glycoprotein-Glycosaminoglycan interactions to inhibit herpes simplex Virus 1 entry and cell-to-cell spread. J. Virol..

[58] Lin L.-T., Chen T.-Y., Lin S.-C., Chung C.-Y., Lin T.-C., Wang G.-H., Anderson R., Lin C.-C., Richardson C.D. (2013). Broad-spectrum antiviral activity of chebulagic acid and punicalagin against viruses that use glycosaminoglycans for entry. BMC Microbiol..

[59] Blanco E., Sabetta W., Danzi D., Negro D., Passeri V., De Lisi A., Paolocci F., Sonnante G. (2018). Isolation and characterization of the flavonol regulator CcMYB12 From the Globe Artichoke [Cynara cardunculus var. scolymus (L.) Fiori]. Front. Plant Sci..

[60] Ravishankar D., Rajora A.K., Greco F., Osborn H.M.I. (2013). Flavonoids as prospective compounds for anti-cancer therapy. Int. J. Biochem. Cell Biol..

[61] Airoldi C., La Ferla B., D‘Orazio G., Ciaramelli C., Palmioli A. (2018). Flavonoids in the treatment of Alzheimer’s and other neurodegenerative diseases. Curr. Med. Chem..

[62] Ding Y., Li C., Zhang Y., Ma P., Zhao T., Che D., Cao J., Wang J., Liu R., Zhang T. (2020). Quercetin as a Lyn kinase inhibitor inhibits IgE-mediated allergic conjunctivitis. Food Chem. Toxicol..

[63] Goh F.Y., Upton N., Guan S., Cheng C., Shanmugam M.K., Sethi G., Leung B.P., Wong W.S.F. (2012). Fisetin, a bioactive flavonol, attenuates allergic airway inflammation through negative regulation of NF-κB. Eur. J. Pharmacol..

[64] Cui J., Wang G., Kandhare A.D., Mukherjee-Kandhare A.A., Bodhankar S.L. (2018). Neuroprotective effect of naringin, a flavone glycoside in quinolinic acid-induced neurotoxicity: Possible role of PPAR-γ, Bax/Bcl-2, and caspase-3. Food Chem. Toxicol..

[65] Pingili R.B., Challa S.R., Pawar A.K., Toleti V., Kodali T., Koppula S. (2020). A systematic review on hepatoprotective activity of quercetin against various drugs and toxic agents: Evidence from preclinical studies. Phyther. Res..

[66] Vicente-Vicente L., González-Calle D., Casanova A.G., Hernández-Sánchez M.T., Prieto M., Rama-Merchán J.C., Martín-Moreiras J., Martín-Herrero F., Sánchez P.L., López-Hernández F.J. (2019). Quercetin, a promising clinical candidate for the prevention of contrast-induced Nephropathy. Int. J. Mol. Sci..

[67] Ezzati M., Yousefi B., Velaei K., Safa A. (2020). A review on anti-cancer properties of Quercetin in breast cancer. Life Sci..

[68] Wang S., Yao J., Zhou B., Yang J., Chaudry M.T., Wang M., Xiao F., Li Y., Yin W. (2018). Bacteriostatic effect of Quercetin as an Antibiotic Alternative in vivo and its antibacterial mechanism in vitro. J. Food Prot..

[69] Pal A., Tripathi A. (2019). Quercetin potentiates meropenem activity among pathogenic carbapenem-resistant *Pseudomonas aeruginosa* and *Acinetobacter baumannii*. J. Appl. Microbiol..

[70] Pendota S.C., Aderogba M.A., Ndhlala A.R., Van Staden J. (2013). Antimicrobial and acetylcholinesterase inhibitory activities of Buddleja salviifolia (L.) Lam. leaf extracts and isolated compounds. J. Ethnopharmacol..

[71] Chattopadhyay D., Naik T. (2007). Antivirals of ethnomedicinal origin: Structure-activity relationship and scope. Mini-Reviews Med. Chem..

[72] Huang H.-C., Tao M.-H., Hung T.-M., Chen J.-C., Lin Z.-J., Huang C. (2014). (−)-Epigallocatechin-3-gallate inhibits entry of hepatitis B virus into hepatocytes. Antivir. Res..

[73] Chiow K.H., Phoon M.C., Putti T., Tan B.K.H., Chow V.T. (2016). Evaluation of antiviral activities of Houttuynia cordata Thunb. extract, quercetin, quercetrin and cinanserin on murine coronavirus and dengue virus infection. Asian Pac. J. Trop. Med..

[74] Yao C., Xi C., Hu K., Gao W., Cai X., Qin J., Lv S., Du C., Wei Y. (2018). Inhibition of enterovirus 71 replication and viral 3C protease by quercetin. Virol. J..

[75] De González-Búrquez M.J., González-Díaz F.R., García-Tovar C.G., Carrillo-Miranda L., Soto-Zárate C.I., Canales-Martínez M.M., Penieres-Carrillo J.G., Crúz-Sánchez T.A., Fonseca-Coronado S. (2018). Comparison between in vitro antiviral effect of Mexican propolis and three commercial Flavonoids against Canine Distemper Virus. Evid. Based Complement. Altern. Med..

[76] Salehi B., Fokou P., Sharifi-Rad M., Zucca P., Pezzani R., Martins N., Sharifi-Rad J. (2019). The therapeutic potential of Naringenin: A review of clinical trials. Pharmaceuticals.

[77] Choi J.-G., Lee H., Kim Y.S., Hwang Y.-H., Oh Y.-C., Lee B., Moon K.M., Cho W.-K., Ma J.Y. (2019). *Aloe vera* and its components inhibit influenza a virus-induced autophagy and replication. Am. J. Chin. Med..

[78] Marunaka Y. (2017). Actions of quercetin, a flavonoid, on ion transporters: Its physiological roles. Ann. N. Y. Acad. Sci..

[79] Lin Y.-J., Chang Y.-C., Hsiao N.-W., Hsieh J.-L., Wang C.-Y., Kung S.-H., Tsai F.-J., Lan Y.-C., Lin C.-W. (2012). Fisetin and rutin as 3C protease inhibitors of enterovirus A71. J. Virol. Methods.

[80] Özçelik B., Kartal M., Orhan I. (2011). Cytotoxicity, antiviral and antimicrobial activities of alkaloids, flavonoids, and phenolic acids. Pharm. Biol..

[81] He W. (2011). Epigallocatechin gallate inhibits HBV DNA synthesis in a viral replication—inducible cell line. World J. Gastroenterol..

[82] Zhong L., Hu J., Shu W., Gao B., Xiong S. (2015). Epigallocatechin-3-gallate opposes HBV-induced incomplete autophagy by enhancing lysosomal acidification, which is unfavorable for HBV replication. Cell Death Dis..

[83] Colpitts C.C., Schang L.M. (2014). A Small molecule inhibits virion attachment to Heparan Sulfate- or Sialic Acid-containing Glycans. J. Virol..

[84] Xu J., Xu Z., Zheng W. (2017). A review of the antiviral role of green tea catechins. Molecules.

[85] Jasso-Miranda C., Herrera-Camacho I., Flores-Mendoza L.K., Dominguez F., Vallejo-Ruiz V., Sanchez-Burgos G.G., Pando-Robles V., Santos-Lopez G., Reyes-Leyva J. (2019). Antiviral and immunomodulatory effects of polyphenols on macrophages infected with dengue virus serotypes 2 and 3 enhanced or not with antibodies. Infect. Drug Resist..

[86] Igbe I., Shen X.-F., Jiao W., Qiang Z., Deng T., Li S., Liu W.-L., Liu H.-W., Zhang G.-L., Wang F. (2017). Dietary quercetin potentiates the antiproliferative effect of interferon-α in hepatocellular carcinoma cells through activation of JAK/STAT pathway signaling by inhibition of SHP2 phosphatase. Oncotarget.

[87] Trujillo-Correa A.I., Quintero-Gil D.C., Diaz-Castillo F., Quiñones W., Robledo S.M., Martinez-Gutierrez M. (2019). In vitro and in silico anti-dengue activity of compounds obtained from Psidium guajava through bioprospecting. BMC Complement. Altern. Med..

[88] Dwivedi V.D., Bharadwaj S., Afroz S., Khan N., Ansari M.A., Yadava U., Tripathi R.C., Tripathi I.P., Mishra S.K., Kang S.G. (2020). Anti-dengue infectivity evaluation of bioflavonoid from *Azadirachta indica* by dengue virus serine protease inhibition. J. Biomol. Struct. Dyn..

[89] Ismail N.A., Jusoh S.A. (2017). Molecular docking and molecular dynamics simulation studies to predict flavonoid binding on the surface of DENV2 E protein. Interdiscip. Sci. Comput. Life Sci..

[90] Qamar M., Mumtaz A., Naseem R., Ali A., Fatima T., Jabbar T., Ahmad Z., Ashfaq U.A. (2014). Molecular docking based screening of plant flavonoids as Dengue NS1 inhibitors. Bioinformation.

[91] De Sousa L.R.F., Wu H., Nebo L., Fernandes J.B., das Graças Fernandes da Silva M.F., Kiefer W., Kanitz M., Bodem J., Diederich W.E., Schirmeister T. (2015). Flavonoids as noncompetitive inhibitors of Dengue virus NS2B-NS3 protease: Inhibition kinetics and docking studies. Bioorg. Med. Chem..

[92] Senthilvel P., Lavanya P., Kumar K.M., Swetha R., Anitha P., Bag S., Sarveswari S., Vijayakumar V., Ramaiah S., Anbarasu A. (2013). Flavonoid from Carica papaya inhibits NS2B-NS3 protease and prevents Dengue 2 viral assembly. Bioinformation.

[93] Chappell K., Stoermer M., Fairlie D., Young P. (2008). West Nile Virus NS2B/NS3 protease as an antiviral target. Curr. Med. Chem..

[94] Kim Y.M., Gayen S., Kang C., Joy J., Huang Q., Chen A.S., Wee J.L.K., Ang M.J.Y., Lim H.A., Hung A.W. (2013). NMR Analysis of a novel enzymatically active unlinked Dengue NS2B-NS3 protease complex. J. Biol. Chem..

[95] Keivan Z., Teoh B.-T., Sam S.-S., Wong P.-F., Mustafa M.R., AbuBakar S. (2011). In vitro antiviral activity of fisetin, rutin and naringenin against dengue virus type-2. J. Med. Plants Res..

[96] Zandi K., Teoh B.-T., Sam S.-S., Wong P.-F., Mustafa M., AbuBakar S. (2011). Antiviral activity of four types of bioflavonoid against dengue virus type-2. Virol. J..

[97] Raekiansyah M., Buerano C.C., Luz M.A.D., Morita K. (2018). Inhibitory effect of the green tea molecule EGCG against dengue virus infection. Arch. Virol..

[98] Vázquez-Calvo Á., Jiménez de Oya N., Martín-Acebes M.A., Garcia-Moruno E., Saiz J.-C. (2017). Antiviral properties of the natural Polyphenols Delphinidin and Epigallocatechin Gallate against the Flaviviruses West Nile Virus, Zika Virus, and Dengue Virus. Front. Microbiol..

[99] Johari J., Kianmehr A., Mustafa M., Abubakar S., Zandi K. (2012). Antiviral activity of Baicalein and Quercetin against the Japanese Encephalitis Virus. Int. J. Mol. Sci..

[100] Zandi K., Teoh B.-T., Sam S.-S., Wong P.-F., Mustafa M.R., AbuBakar S. (2012). Novel antiviral activity of baicalein against dengue virus. BMC Complement. Altern. Med..

[101] Shukla Y., Singh R. (2011). Resveratrol and cellular mechanisms of cancer prevention. Ann. N. Y. Acad. Sci..

[102] Shen T., Wang X.-N., Lou H.-X. (2009). Natural stilbenes: An overview. Nat. Prod. Rep..

[103] Ito T. (2020). Resveratrol oligomer structure in Dipterocarpaceaeous plants. J. Nat. Med..

[104] Nawaz W., Zhou Z., Deng S., Ma X., Ma X., Li C., Shu X. (2017). Therapeutic versatility of resveratrol derivatives. Nutrients.

[105] Thapa S.B., Pandey R.P., Park Y., Sohng J.K. (2019). Biotechnological advances in resveratrol production and its chemical diversity. Molecules.

[106] Abba Y., Hassim H., Hamzah H., Noordin M.M. (2015). Antiviral activity of resveratrol against human and animal viruses. Adv. Virol..

[107] Mohd A., Zainal N., Tan K.-K., AbuBakar S. (2019). Resveratrol affects Zika virus replication in vitro. Sci. Rep..

[108] Nakamura M. (2010). An antioxidant resveratrol significantly enhanced replication of hepatitis C virus. World J. Gastroenterol..

[109] Paemanee A., Hitakarun A., Roytrakul S., Smith D.R. (2018). Screening of melatonin, α-tocopherol, folic acid, acetyl-l-carnitine and resveratrol for anti-dengue 2 virus activity. BMC Res. Notes.

[110] Zainal N., Chang C.-P., Cheng Y.-L., Wu Y.-W., Anderson R., Wan S.-W., Chen C.-L., Ho T.-S., AbuBakar S., Lin Y.-S. (2017). Resveratrol treatment reveals a novel role for HMGB1 in regulation of the type 1 interferon response in dengue virus infection. Sci. Rep..

[111] Ong S.P., Lee L.M., Leong Y.F.I., Ng M.L., Chu J.J.H. (2012). Dengue virus infection mediates HMGB1 release from monocytes involving PCAF acetylase complex and induces vascular leakage in Endothelial cells. PLoS ONE.

[112] Han Y.-S., Penthala N.R., Oliveira M., Mesplède T., Xu H., Quan Y., Crooks P.A., Wainberg M.A. (2017). Identification of resveratrol analogs as potent anti-dengue agents using a cell-based assay. J. Med. Virol..

[113] Krishnan M., Garcia-Blanco M. (2014). Targeting host factors to treat west Nile and Dengue Viral infections. Viruses.

[114] De Wispelaere M., LaCroix A.J., Yang P.L. (2013). The Small Molecules AZD0530 and Dasatinib Inhibit Dengue Virus RNA Replication via Fyn Kinase. J. Virol..

[115] Lü J.-M., Nurko J., Weakley S.M., Jiang J., Kougias P., Lin P.H., Yao Q., Chen C. (2010). Molecular mechanisms and clinical applications of nordihydroguaiaretic acid (NDGA) and its derivatives: An update. Med. Sci. Monit. Int. Med. J. Exp. Clin. Res..

[116] Manda G., Rojo A.I., Martínez-Klimova E., Pedraza-Chaverri J., Cuadrado A. (2020). Nordihydroguaiaretic Acid: From herbal medicine to clinical development for cancer and chronic diseases. Front. Pharmacol..

[117] Hernández-Damián J., Andérica-Romero A.C., Pedraza-Chaverri J. (2014). Paradoxical cellular effects and biological role of the multifaceted compound Nordihydroguaiaretic Acid. Arch. Pharm. (Weinheim).

[118] Iranpour M., Moghadam A.R., Yazdi M., Ande S.R., Alizadeh J., Wiechec E., Lindsay R., Drebot M., Coombs K.M., Ghavami S. (2016). Apoptosis, autophagy and unfolded protein response pathways in Arbovirus replication and pathogenesis. Expert Rev. Mol. Med..

[119] Hwu J.R., Hsu M.-H., Huang R.C.C. (2008). New nordihydroguaiaretic acid derivatives as anti-HIV agents. Bioorg. Med. Chem. Lett..

[120] Uchide N., Ohyama K., Bessho T., Toyoda H. (2005). Inhibition of Influenza-Virus-Induced Apoptosis in Chorion cells of human fetal membranes by Nordihydroguaiaretic Acid. Intervirology.

[121] Syed G.H., Siddiqui A. (2011). Effects of hypolipidemic agent nordihydroguaiaretic acid on lipid droplets and hepatitis C virus. Hepatology.

[122] Samsa M.M., Mondotte J.A., Iglesias N.G., Assunção-Miranda I., Barbosa-Lima G., Da Poian A.T., Bozza P.T., Gamarnik A.V. (2009). Dengue virus Capsid protein usurps lipid droplets for viral particle formation. PLoS Pathog..

[123] Soto-Acosta R., Bautista-Carbajal P., Syed G.H., Siddiqui A., Del Angel R.M. (2014). Nordihydroguaiaretic acid (NDGA) inhibits replication and viral morphogenesis of Dengue virus. Antivir. Res..

[124] Carvalho F.A., Carneiro F.A., Martins I.C., Assuncao-Miranda I., Faustino A.F., Pereira R.M., Bozza P.T., Castanho M.A.R.B., Mohana-Borges R., Da Poian A.T. (2012). Dengue Virus Capsid protein binding to Hepatic Lipid Droplets (LD) is Potassium Ion dependent and is mediated by LD surface proteins. J. Virol..

[125] Martínez-Gutierrez M., Castellanos J.E., Gallego-Gómez J.C. (2011). Statins reduce Dengue virus production via decreased Virion assembly. Intervirology.

[126] Martín-Acebes M.A., Vázquez-Calvo Á., Saiz J.-C. (2016). Lipids and flaviviruses, present and future perspectives for the control of dengue, Zika, and West Nile viruses. Prog. Lipid Res..

[127] Villareal V.A., Rodgers M.A., Costello D.A., Yang P.L. (2015). Targeting host lipid synthesis and metabolism to inhibit dengue and hepatitis C viruses. Antivir. Res..

[128] Merino-Ramos T., Blázquez A.-B., Escribano-Romero E., Cañas-Arranz R., Sobrino F., Saiz J.-C., Martín-Acebes M.A. (2014). Protection of a single dose West Nile Virus recombinant subviral particle vaccine against Lineage 1 or 2 strains and analysis of the cross-reactivity with Usutu virus. PLoS ONE.

[129] Manolova Y., Deneva V., Antonov L., Drakalska E., Momekova D., Lambov N. (2014). The effect of the water on the curcumin tautomerism: A quantitative approach. Spectrochim. Acta Part A Mol. Biomol. Spectrosc..

[130] Katsuyama Y., Kita T., Funa N., Horinouchi S. (2009). Curcuminoid Biosynthesis by two type III Polyketide Synthases in the Herb *Curcuma longa*. J. Biol. Chem..

[131] Kocaadam B., Şanlier N. (2017). Curcumin, an active component of turmeric ( *Curcuma longa* ), and its effects on health. Crit. Rev. Food Sci. Nutr..

[132] Joe B., Vijaykumar M., Lokesh B.R. (2004). Biological properties of curcumin-cellular and molecular mechanisms of action. Crit. Rev. Food Sci. Nutr..

[133] Ingolfsson H.I., Koeppe R.E., Andersen O.S. (2007). Curcumin is a modulator of Bilayer material properties ^†^. Biochemistry.

[134] Si X., Wang Y., Wong J., Zhang J., McManus B.M., Luo H. (2007). Dysregulation of the Ubiquitin-Proteasome system by curcumin suppresses Coxsackievirus B3 replication. J. Virol..

[135] Dutta K., Ghosh D., Basu A. (2009). Curcumin protects neuronal cells from Japanese Encephalitis Virus-mediated cell death and also inhibits infective viral particle formation by Dysregulation of Ubiquitin–Proteasome system. J. Neuroimmune Pharmacol..

[136] Kutluay S.B., Doroghazi J., Roemer M.E., Triezenberg S.J. (2008). Curcumin inhibits herpes simplex virus immediate-early gene expression by a mechanism independent of p300/CBP histone acetyltransferase activity. Virology.

[137] Lin X., Ammosova T., Kumari N., Nekhai S. (2017). Protein Phosphatase-1 –targeted small molecules, Iron Chelators and Curcumin analogs as HIV-1 antivirals. Curr. Pharm. Des..

[138] Anggakusuma, Colpitts C.C., Schang L.M., Rachmawati H., Frentzen A., Pfaender S., Behrendt P., Brown R.J.P., Bankwitz D., Steinmann J. (2014). Turmeric curcumin inhibits entry of all hepatitis C virus genotypes into human liver cells. Gut.

[139] Mounce B.C., Cesaro T., Carrau L., Vallet T., Vignuzzi M. (2017). Curcumin inhibits Zika and chikungunya virus infection by inhibiting cell binding. Antivir. Res..

[140] Fernandez-Garcia M.-D., Meertens L., Bonazzi M., Cossart P., Arenzana-Seisdedos F., Amara A. (2011). Appraising the roles of CBLL1 and the Ubiquitin/Proteasome system for Flavivirus entry and replication. J. Virol..

[141] Padilla-S L., Rodríguez A., Gonzales M.M., Gallego-G J.C., Castaño-O J.C. (2014). Inhibitory effects of curcumin on dengue virus type 2-infected cells in vitro. Arch. Virol..

[142] Chen T.-Y., Chen D.-Y., Wen H.-W., Ou J.-L., Chiou S.-S., Chen J.-M., Wong M.-L., Hsu W.-L. (2013). Inhibition of enveloped viruses infectivity by Curcumin. PLoS ONE.

[143] Nguyen T.T.H., Si J., Kang C., Chung B., Chung D., Kim D. (2017). Facile preparation of water soluble curcuminoids extracted from turmeric ( Curcuma longa L.) powder by using steviol glucosides. Food Chem..

[144] Torrens-Spence M.P., Pluskal T., Li F.-S., Carballo V., Weng J.-K. (2018). Complete pathway elucidation and heterologous reconstitution of Rhodiola Salidroside biosynthesis. Mol. Plant.

[145] Zhong Z., Han J., Zhang J., Xiao Q., Hu J., Chen L. (2018). Pharmacological activities, mechanisms of action, and safety of salidroside in the central nervous system. Drug Des. Devel. Ther..

[146] Sa L., Wei X., Huang Q., Cai Y., Lu D., Mei R., Hu X. (2020). Contribution of salidroside to the relieve of symptom and sign in the early acute stage of osteoarthritis in rat model. J. Ethnopharmacol..

[147] Gao H., Peng L., Li C., Ji Q., Li P. (2020). Salidroside alleviates cartilage degeneration through NF-κB pathway in Osteoarthritis Rats. Drug Des. Devel. Ther..

[148] Shati A.A. (2020). Salidroside ameliorates diabetic nephropathy in rats by activating renal AMPK/SIRT1 signaling pathway. J. Food Biochem..

[149] Sun A., Ju X.-L. (2020). Advances in research on anticancer properties of Salidroside. Chin. J. Integr. Med..

[150] Agbo M.O., Odimegwu D.C., Okoye F.B.C., Osadebe P.O. (2017). Antiviral activity of Salidroside from the leaves of Nigerian mistletoe (Loranthus micranthus Linn) parasitic on Hevea brasiliensis against respiratory syncytial virus. Pak. J. Pharm. Sci..

[151] Wang H., Ding Y., Zhou J., Sun X., Wang S. (2009). The in vitro and in vivo antiviral effects of salidroside from Rhodiola rosea L. against coxsackievirus B3. Phytomedicine.

[152] Cheng Y.-L., Lin Y.-S., Chen C.-L., Wan S.-W., Ou Y.-D., Yu C.-Y., Tsai T.-T., Tseng P.-C., Lin C.-F. (2015). Dengue virus infection causes the activation of distinct NF- *κ* B pathways for inducible Nitric Oxide synthase and TNF- *α* expression in RAW264.7 Cells. Mediators Inflamm..

[153] Lee Y., Jung J.-C., Jang S., Kim J., Ali Z., Khan I.A., Oh S. (2013). Anti-inflammatory and neuroprotective effects of constituents isolated from *Rhodiola rosea*. Evidence-Based Complement. Altern. Med..

[154] Zuo G., Li Z., Chen L., Xu X. (2007). Activity of compounds from Chinese herbal medicine Rhodiola kirilowii (Regel) Maxim against HCV NS3 serine protease. Antivir. Res..

[155] Sharma N., Mishra K.P., Ganju L. (2016). Salidroside exhibits anti-dengue virus activity by upregulating host innate immune factors. Arch. Virol..

[156] Gack M.U. (2014). Mechanisms of RIG-I-Like Receptor activation and manipulation by Viral Pathogens. J. Virol..

[157] Navarro-Sánchez E., Desprès P., Cedillo-Barrón L. (2005). Innate immune responses to Dengue virus. Arch. Med. Res..

[158] Sadler A.J., Williams B.R.G. (2007). Structure and function of the protein Kinase R. Interferon: The 50th Anniversary.

[159] Beltrán D., López-Vergès S. (2014). NK Cells during Dengue disease and their recognition of Dengue virus-infected cells. Front. Immunol..

[160] Martins F.O., Esteves P.F., Mendes G.S., Barbi N.S., Menezes F.S., Romanos M.T. (2009). Verbascoside isolated from Lepechinia speciosa has inhibitory activity against HSV-1 and HSV-2 in vitro. Nat. Prod. Commun..

[161] Alipieva K., Korkina L., Orhan I.E., Georgiev M.I. (2014). Verbascoside—A review of its occurrence, (bio)synthesis and pharmacological significance. Biotechnol. Adv..

[162] Alvarenga T.A., Bertanha C.S., de Oliveira P.F., Tavares D.C., Gimenez V.M.M., Silva M.L.A., Cunha W.R., Januário A.H., Pauletti P.M. (2015). Lipoxygenase inhibitory activity of *Cuspidaria pulchra* and isolated compounds. Nat. Prod. Res..

[163] Brandão G.C., Kroon E.G., dos Santos J.R., Stehmann J.R., Lombardi J.A., Oliveira A.B. (2010). de Antiviral activities of plants occurring in the state of Minas Gerais, Brazil: Part 2. Screening Bignoniaceae species. Rev. Bras. Farmacogn..

[164] Le J., Lu W., Xiong X., Wu Z., Chen W. (2015). Anti-inflammatory constituents from Bidens frondosa. Molecules.

[165] Brandão G., Kroon E., Souza D., Filho J., Oliveira A. (2013). Chemistry and antiviral activity of Arrabidaea pulchra (Bignoniaceae). Molecules.

[166] McDougall B., King P.J., Wu B.W., Hostomsky Z., Reinecke M.G., Robinson W.E. (1998). Dicaffeoylquinic and Dicaffeoyltartaric acids are selective inhibitors of human immunodeficiency virus type 1 integrase. Antimicrob. Agents Chemother..

[167] Ma J.N., Bolraa S., Ji M., He Q.Q., Ma C.M. (2016). Quantification and antioxidant and anti-HCV activities of the constituents from the inflorescences of *Scabiosa comosa* and *S. tschilliensis*. Nat. Prod. Res..

[168] Kopp E., Ghosh S. (1994). Inhibition of NF-kappa B by sodium salicylate and aspirin. Science.

[169] Mitchell J.A., Saunders M., Barnes P.J., Newton R., Belvisi M.G. (1997). Sodium Salicylate inhibits Cyclo-Oxygenase-2 activity independently of transcription factor (nuclear factor κB) activation: Role of arachidonic acid. Mol. Pharmacol..

[170] Bitko V., Velazquez A., Yang L., Yang Y.-C., Barik S. (1997). Transcriptional induction of multiple Cytokines by human respiratory Syncytial Virus requires activation of NF-κB and is inhibited by Sodium Salicylate and aspirin. Virology.

[171] Speir E., Yu Z.-X., Ferrans V.J., Huang E.-S., Epstein S.E. (1998). Aspirin attenuates Cytomegalovirus infectivity and gene expression mediated by Cyclooxygenase-2 in coronary artery smooth muscle cells. Circ. Res..

[172] Chen C.-J., Raung S.-L., Kuo M.-D., Wang Y.-M. (2002). Suppression of Japanese encephalitis virus infection by non-steroidal anti-inflammatory drugs. J. Gen. Virol..

[173] Liao C.-L., Lin Y.-L., Wu B.-C., Tsao C.-H., Wang M.-C., Liu C.-I., Huang Y.-L., Chen J.-H., Wang J.-P., Chen L.-K. (2001). Salicylates inhibit Flavivirus replication independently of blocking nuclear factor Kappa B activation. J. Virol..

[174] Jiang Y., Chen C., Li Z., Guo W., Gegner J.A., Lin S., Han J. (1996). Characterization of the structure and function of a New Mitogen-activated Protein Kinase (p38β). J. Biol. Chem..

[175] Cox D. (2020). Anti-platelet agents: Past, present and future. ISBT Sci. Ser..

[176] Alvarenga T.A., de Oliveira P.F., de Souza J.M., Tavares D.C., Andrade e Silva M.L., Cunha W.R., Groppo M., Januário A.H., Magalhães L.G., Pauletti P.M. (2016). Schistosomicidal Activity of Alkyl-phenols from the Cashew *Anacardium occidentale* against *Schistosoma mansoni* Adult Worms. J. Agric. Food Chem..

[177] Zhuang J.-X., Hu Y.-H., Yang M.-H., Liu F.-J., Qiu L., Zhou X.-W., Chen Q.-X. (2010). Irreversible competitive inhibitory kinetics of Cardol Triene on mushroom Tyrosinase. J. Agric. Food Chem..

[178] Matutino Bastos T., Mannochio Russo H., Silvio Moretti N., Schenkman S., Marcourt L., Gupta M., Wolfender J.-L., Ferreira Queiroz E., Botelho Pereira Soares M. (2019). Chemical constituents of Anacardium occidentale as inhibitors of Trypanosoma cruzi Sirtuins. Molecules.

[179] Kanyaboon P., Saelee T., Suroengrit A., Hengphasatporn K., Rungrotmongkol T., Chavasiri W., Boonyasuppayakorn S. (2018). Cardol triene inhibits dengue infectivity by targeting kl loops and preventing envelope fusion. Sci. Rep..

[180] HERBST K.H. (1959). Albothyl as a hemostatic agent in otorhinolaryngology. Z. Laryngol. Rhinol. Otol..

[181] Silva L.A.F., Fioravanti M.C.S., Oliveira K.S., Atayde I.B., Andrade M.A., Jayme V.S., Rabelo R.E., Romani A.F., Araújo E.G. (2004). Local utilization of Metacresolsulfonic acid combined with Streptomycin in the treatment of Actinomycosis. Ann. N. Y. Acad. Sci..

[182] Ali A., Al-sobayil F.A., Al-Hawas A. (2010). Evaluating the effectiveness of different treatments of uterine infections in female camels (Camelus dromedarius). Theriogenology.

[183] Tomlinson S., Malmstrom R., Watowich S. (2009). New approaches to structure-based discovery of Dengue protease inhibitors. Infect. Disord.-Drug Targets.

[184] Geiss B.J., Stahla H., Hannah A.M., Gari H.H., Keenan S.M. (2009). Focus on flaviviruses: Current and future drug targets. Future Med. Chem..

[185] Wu D., Mao F., Ye Y., Li J., Xu C., Luo X., Chen J., Shen X. (2015). Policresulen, a novel NS2B/NS3 protease inhibitor, effectively inhibits the replication of DENV2 virus in BHK-21 cells. Acta Pharmacol. Sin..

[186] Chanprapaph S., Saparpakorn P., Sangma C., Niyomrattanakit P., Hannongbua S., Angsuthanasombat C., Katzenmeier G. (2005). Competitive inhibition of the dengue virus NS3 serine protease by synthetic peptides representing polyprotein cleavage sites. Biochem. Biophys. Res. Commun..

[187] Johnson K., Liu L., Majdzadeh N., Chavez C., Chin P.C., Morrison B., Wang L., Park J., Chugh P., Chen H.-M. (2005). Inhibition of neuronal apoptosis by the cyclin-dependent kinase inhibitor GW8510: Identification of 3′ substituted indolones as a scaffold for the development of neuroprotective drugs. J. Neurochem..

[188] Lackey K., Cory M., Davis R., Frye S.V., Harris P.A., Hunter R.N., Jung D.K., McDonald O.B., McNutt R.W., Peel M.R. (2000). The discovery of potent cRaf1 kinase inhibitors. Bioorg. Med. Chem. Lett..

[189] Chen H.-M., Wang L., D’Mello S.R. (2008). Inhibition of ATF-3 expression by B-Raf mediates the neuroprotective action of GW5074. J. Neurochem..

[190] Chin P.C., Liu L., Morrison B.E., Siddiq A., Ratan R.R., Bottiglieri T., D’Mello S.R. (2004). The c-Raf inhibitor GW5074 provides neuroprotection invitro and in an animal model of neurodegeneration through a MEK-ERK and Akt-independent mechanism. J. Neurochem..

[191] Opoku-Temeng C., Onyedibe K.I., Aryal U.K., Sintim H.O. (2019). Proteomic analysis of bacterial response to a 4-hydroxybenzylidene indolinone compound, which re-sensitizes bacteria to traditional antibiotics. J. Proteom..

[192] Pfleiderer P., Sumandea M.P., Rybin V.O., Wang C., Steinberg S.F. (2009). Raf-1: A novel cardiac troponin T kinase. J. Muscle Res. Cell Motil..

[193] Arita M., Wakita T., Shimizu H. (2008). Characterization of pharmacologically active compounds that inhibit poliovirus and enterovirus 71 infectivity. J. Gen. Virol..

[194] Yang S., Atkinson S., Fraser J., Wang C., Maher B., Roman N., Forwood J., Wagstaff K., Borg N., Jans D. (2019). Novel Flavivirus antiviral that targets the host nuclear transport importin α/β1 heterodimer. Cells.

[195] Lee Y.-J., Lee Y.M., Lee C.-K., Jung J.K., Han S.B., Hong J.T. (2011). Therapeutic applications of compounds in the Magnolia family. Pharmacol. Ther..

[196] Zhang P., Liu X., Zhu Y., Chen S., Zhou D., Wang Y. (2013). Honokiol inhibits the inflammatory reaction during cerebral ischemia reperfusion by suppressing NF-κB activation and cytokine production of glial cells. Neurosci. Lett..

[197] Hu H., Zhang X., Wang Y., Chen S. (2005). Honokiol inhibits arterial thrombosis through endothelial cell protection and stimulation of prostacyclin. Acta Pharmacol. Sin..

[198] Shen J.-L., Man K.-M., Huang P.-H., Chen W.-C., Chen D.-C., Cheng Y.-W., Liu P.-L., Chou M.-C., Chen Y.-H. (2010). Honokiol and Magnolol as multifunctional antioxidative molecules for dermatologic disorders. Molecules.

[199] Liu Y., Cheng P., Wu A.-H. (2020). Honokiol inhibits carotid artery atherosclerotic plaque formation by suppressing inflammation and oxidative stress. Aging.

[200] Talarek S., Listos J., Barreca D., Tellone E., Sureda A., Nabavi S.F., Braidy N., Nabavi S.M. (2017). Neuroprotective effects of honokiol: From chemistry to medicine. BioFactors.

[201] Hahm E.-R., Arlotti J.A., Marynowski S.W., Singh S.V. (2008). Honokiol, a constituent of oriental medicinal herb Magnolia officinalis, inhibits growth of PC-3 Xenografts in vivo in association with Apoptosis induction. Clin. Cancer Res..

[202] Bai X., Cerimele F., Ushio-Fukai M., Waqas M., Campbell P.M., Govindarajan B., Der C.J., Battle T., Frank D.A., Ye K. (2003). Honokiol, a small molecular weight natural product, inhibits Angiogenesis in vitro and tumor growth in Vivo. J. Biol. Chem..

[203] Fried L.E., Arbiser J.L. (2009). Honokiol, a multifunctional antiangiogenic and antitumor agent. Antioxid. Redox Signal..

[204] Kim S.Y., Kim J., Jeong S.-I., Jahng K.Y., Yu K.-Y. (2015). Antimicrobial effects and resistant regulation of Magnolol and Honokiol on Methicillin-resistant *Staphylococcus aureus*. Biomed Res. Int..

[205] Liu S., Li L., Tan L., Liang X. (2019). Inhibition of herpes simplex Virus-1 replication by natural compound Honokiol. Virol. Sin..

[206] Lan K.-H., Wang Y.-W., Lee W.-P., Lan K.-L., Tseng S.-H., Hung L.-R., Yen S.-H., Lin H.-C., Lee S.-D. (2012). Multiple effects of honokiol on the life cycle of hepatitis C virus. Liver Int..

[207] Fang C.-Y., Chen S.-J., Wu H.-N., Ping Y.-H., Lin C.-Y., Shiuan D., Chen C.-L., Lee Y.-R., Huang K.-J. (2015). Honokiol, a lignan Biphenol derived from the Magnolia tree, inhibits Dengue virus type 2 infection. Viruses.

